# A human proteogenomic-cellular framework identifies KIF5A as a modulator of astrocyte process integrity with relevance to ALS

**DOI:** 10.1038/s42003-023-05041-4

**Published:** 2023-06-29

**Authors:** Kornélia Szebényi, Inigo Barrio-Hernandez, George M. Gibbons, Luca Biasetti, Claire Troakes, Pedro Beltrao, András Lakatos

**Affiliations:** 1 John van Geest Centre for Brain Repair, Department of Clinical Neurosciences, University of Cambridge, Cambridge Biomedical Campus, Cambridge, CB2 0PY UK; 2 Institute of Enzymology, Research Centre for Natural Sciences, Budapest, 1117 Hungary; 3 European Bioinformatics Institute, Wellcome Genome Campus, Hinxton, CB10 1SD UK; 4 Department of Basic and Clinical Neuroscience, Institute of Psychiatry, Psychology and Neuroscience, King’s College London, London, SE5 8AF UK; 5 Institute of Molecular Systems Biology, ETH Zürich, Zürich, 8093 Switzerland; 6 Wellcome Trust-MRC Cambridge Stem Cell Institute, Cambridge Biomedical Campus, Cambridge, CB2 0AW UK

**Keywords:** Astrocyte, Mechanisms of disease

## Abstract

Genome-wide association studies identified several disease-causing mutations in neurodegenerative diseases, including amyotrophic lateral sclerosis (ALS). However, the contribution of genetic variants to pathway disturbances and their cell type-specific variations, especially in glia, is poorly understood. We integrated ALS GWAS-linked gene networks with human astrocyte-specific multi-omics datasets to elucidate pathognomonic signatures. It predicts that KIF5A, a motor protein kinesin-1 heavy-chain isoform, previously detected only in neurons, can also potentiate disease pathways in astrocytes. Using postmortem tissue and super-resolution structured illumination microscopy in cell-based perturbation platforms, we provide evidence that KIF5A is present in astrocyte processes and its deficiency disrupts structural integrity and mitochondrial transport. We show that this may underly cytoskeletal and trafficking changes in SOD1 ALS astrocytes characterised by low KIF5A levels, which can be rescued by c-Jun N-terminal Kinase-1 (JNK1), a kinesin transport regulator. Altogether, our pipeline reveals a mechanism controlling astrocyte process integrity, a pre-requisite for synapse maintenance and suggests a targetable loss-of-function in ALS.

## Abstract (web summary)

Interrogation of amyotrophic lateral sclerosis (ALS)-linked GWAS gene networks, astrocyte-based multi-omics datasets and cellular models identifies kinesin motor protein KIF5A as a modifier of astrocyte process integrity and potential target in ALS.

## Introduction

Although human genome-wide association studies (GWAS) have successfully identified genetic variants associated with the development of neurodegenerative diseases, it is still poorly understood how these variants exert their effects on disease risk. Data from GWAS are typically assessed in a single nucleotide polymorphism (SNP) by SNP manner without the consideration of the joint effects of multiple functionally related genes^1–3^. In addition, natural variation in gene expression in individuals may also influence pathway manifestation evoked by single genetic variants^2,4^, and the effects are seldom validated across multiple cell types contributing to pathogenesis^5^. Therefore, to develop more effective therapeutic strategies, we need to understand the relevance of identified associations in various cell types, using better predictive models. In this regard, network-based expansion of GWAS-linked genes has shown promise in identifying such biological pathways^6–9^. This is based on projecting GWAS-linked genes onto a human protein-protein interaction (PPI) network, in which interacting partners are ranked as trait-associated genes. Such network propagation strategies have been demonstrated as powerful methods for drug target identification^10^, highlighting the sufficient biologically relevant predictive power of these algorithms.

Elucidating the effects of associated genetic variants and establishing their cell type-specificity is especially relevant in amyotrophic lateral sclerosis (ALS), where there are currently no effective treatments. ALS is a fatal neurodegenerative disease in which cortical and spinal motor neurons and glial cells, such as astrocytes, are primarily affected, resulting in limb and respiratory muscle paralysis^11^. It is still unresolved how astrocytes trigger and propagate pathology. There is growing evidence for both ALS-causing mutation-driven astrocytic toxic gain of function and loss of function^12^, proposing their key involvement in neuronal pathogenesis. Therefore, it is relevant to address how astrocytes are affected by a combination of genetic variants of different frequencies, potentially enhancing their susceptibility to pathogenic pathways. Since observations of astrocytic roles can often be obscured by their adaptive reactive response to neuronal insults^13^, the combination of using complex datasets for unbiased computational predictions and mechanistic validations in culture-based platforms could enhance discovery.

Here we demonstrate a proteogenomic-cell biology pipeline relevant to exploring ALS pathobiology with a focus on astrocytic signatures. First, we exploit a GWAS network propagation approach to define gene modules enriched in genetic drivers, followed by its integration with our published SOD1 ALS astrocyte-specific transcriptomic and proteomic datasets^14^. In particular, this indicates a motor protein kinesin-1 heavy-chain isoform 5 A (KIF5A) expression defect, which also overlaps with disease-driving gene modules identified by GWAS, implying its key involvement in ALS pathways. While kinesin-1/KIF5A dysfunction has recently been identified in KIF5A and other ALS-causing mutations^15,16^, its presence and role have not been demonstrated in cell types other than neurons in the central nervous system^17,18^. Thus, to explore the unknown function of KIF5A in astrocytes and its potential role in ALS, we utilised perturbation experiments in combination with cytoskeletal analyses, biochemical assays and super-resolution structured illumination microscopy (SR-SIM) in human induced pluripotent stem cell (iPSC)-derived and mouse astrocyte culture platforms. We show that reduced KIF5A levels negatively affect process formation, mitochondrial trafficking and its potential association with glutamate transporter EAAT2. While low KIF5A-expressing SOD1 ALS astrocytes mirror this phenotype, it can be reversed by a kinesin regulator. Our work provides a multi-dimensional framework for a cell type-specific mechanistic discovery process, which could inform therapeutic strategies for ALS and related neurodegenerative disorders.

## Results

### Network expansion of GWAS-identified genes implicates key biological processes across neurodegenerative diseases

**Fig. 1:**
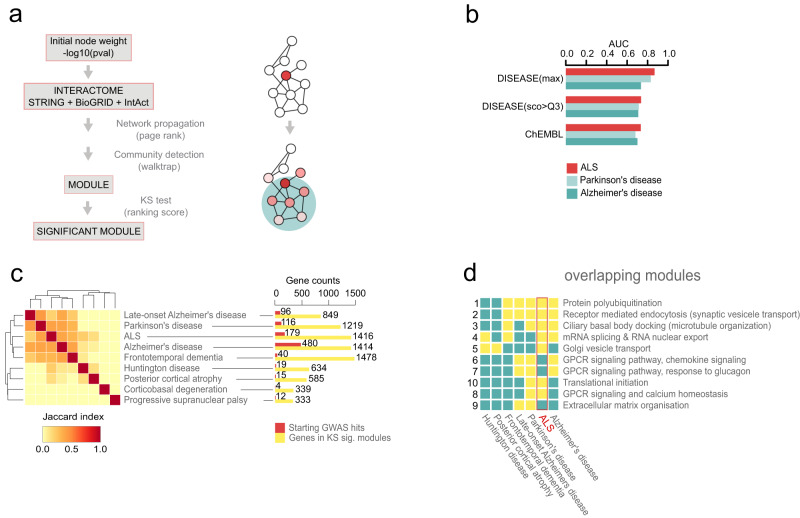
A network-based approach predicts common pathway disruptions in neurodegenerative diseases. **a** Schematic representation of network propagation by mapping GWAS hits onto protein-protein interactions (left), leading to network modules (right). **b** Method benchmarking using gene-to-disease associations extracted from the DISEASE database and drug targets from ChEMBL datasets. Barplots represent the areas under the receiver operator characteristic (ROC) curves (AUCs) for three conditions, ALS, Parkinson’s and Alzheimer’s diseases. **c** Heatmap represents gene overlaps between various neurodegenerative disease modules, based on the Jaccard similarity index. Barplots illustrate the gene counts from the initial GWAS input (red) and from the expanded significant modules used for Jaccard index calculations (yellow). **d** Heatmap showing overlaps between the 10 gene-network modules, shared amongst at least two neurodegenerative diseases (Jaccard index >0.7, yellow colour), and described using Gene Ontology Biological Process (GOBP) annotation (right sided list). Please, also see Suppl. Figure 1.

To achieve unbiased predictions of affected biological pathways in neurodegenerative diseases, we developed an analysis framework, based on a network expansion method. First, we compiled a comprehensive human interactome by combining high-confidence data from multiple databases, resulting in 540,421 interactions between 18,055 proteins. Next, using the GWAS catalogue, we extracted all genes mapping close to single nucleotide polymorphisms (SNPs) significantly associated with one or more of nine neurodegenerative diseases, including ALS. We then applied a network-based approach to identify a set of human proteins that are highly connected with risk genes (Fig. 1a). To confirm the value of this method, we defined two gold-standard gene-to-disease association sets. Genes linked to neurodegenerative diseases were obtained from the DISEASE database^19^ (http://diseases.jensenlab.org) and segregated into two groups according to the degree of confidence and known targets of drugs from studies extracted from ChEMBL (https://www.ebi.ac.uk/chembl). We benchmarked the predictive power of identifying these genes by their network propagation score, calculated by the area under the receiver operating curve (ROC). The average score was higher than 0.7 in the two gold standard sets (Fig. 1b), indicating a strong predictive power.

To identify protein communities or modules with disease association, we defined highly interconnected proteins by clustering the interaction network. Protein communities enriched for disease genes were identified by network propagation and labelled as disease-associated gene modules. Next, we explored common pathways involved in the selected neurodegenerative diseases by identifying gene overlaps from all significant modules using the Jaccard index (Fig. 1c). Alzheimer’s disease, Parkinson’s disease, ALS, and frontotemporal dementia were clustered together, suggesting a higher number of genetic commonalities. Huntington’s disease, posterior cortical atrophy, corticobasal degeneration, and progressive supranuclear palsy appeared to be unique, although the smaller number of GWAS hits in these cases may have been a confounding factor. The three modules shared across most diseases, including ALS, were linked to protein ubiquitination, receptor-mediated endocytosis including synaptic vesicle transport, and microtubule organisation (Fig. 1d, Supplementary Fig. 1a). These biological entities are represented in the converging pathways of neurodegenerative diseases^20^. Although the individual GWAS-linked genes related to these biological processes do not strictly overlap, they share protein interaction partners, illustrating the value of network propagation in the discovery of common and specific pathogenic routes. Overall, our computational prediction well reflected the previously described commonalities in pathogenic pathways but also pointed out the divergence of specific genes linked to cellular processes in various neurodegenerative diseases. Therefore, our network-based method is well-suited to propose pathways predisposing to neurodegenerative pathogenesis.

### Network-based integration of GWAS with astrocyte-specific multi-omics data predicts susceptibility genes

**Fig. 2:**
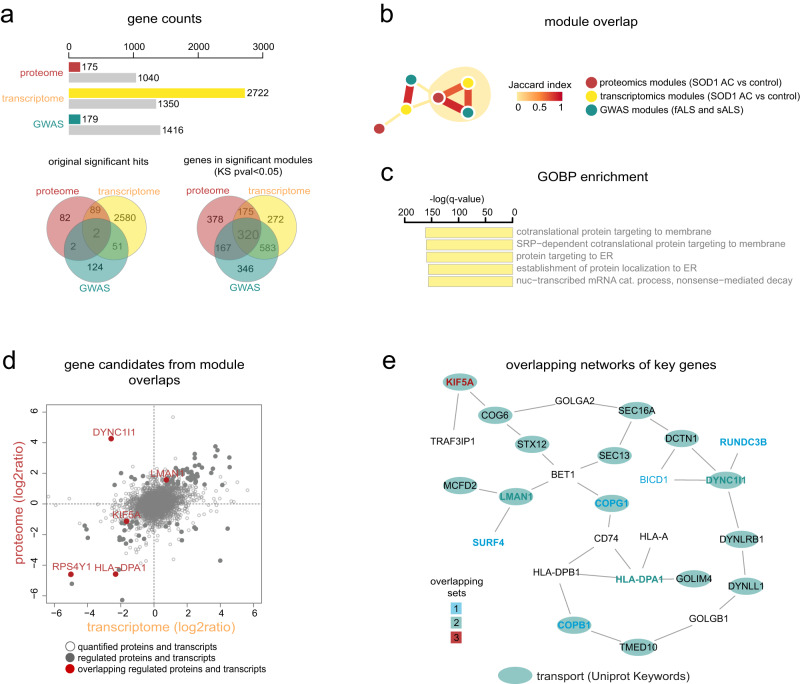
Integration of multi-omics datasets predicts KIF5A-related pathway disturbances. **a** Protein (red), transcript (yellow) and gene (green) counts representing the input elements and the number of elements following network-expansion (grey). Venn diagrams (lower panel) illustrate the number of overlapping elements between the significant proteome, transcriptome and GWAS modules for the initial input (left) and for the network-expanded dataset (right). **b** Network illustrating the top significant module overlap (nodes). The edge thickness and colours illustrate the number of shared genes between modules as defined by the Jaccard index. **c** Barplots demonstrate the Gene Ontology Biological Process (GOBP) enrichment results for the genes included in the triple overlap (Fisher test, Benjamini-Hochberg adjusted *p* value < 0.05). **d** Scatter plot showing the log2 ratios for the changes in the proteomic and transcriptomic datasets (grey dots), amongst which the red dots illustrate genes overlapping with significant GWAS modules (b). **e** Shortest path interactome between the overlapping candidate genes and their first-degree network neighbours. The text colours correspond with the number of sets that share the given gene, and the green coloured nodes represent genes associated with intracellular transport function as indicated (Uniprot Keyword annotation). Please, also see Suppl. Figure 2.

To explore astrocyte-specific aspects of predisposing pathways to ALS pathogenesis, we integrated our GWAS network-based modules with our published transcriptomic and proteomic datasets deriving from human SOD1 ALS patient-specific iPSC-derived astrocytes^14^. For this, we used genes from the GWAS network modules (*n* = 179), differentially expressed transcripts (*n* = 2,772) and altered protein levels (*n* = 175) between SOD1-mutant and control ALS astrocytes, including genetically corrected controls. Our network expansion-based analysis, starting with each of the three datasets that identified 320 overlapping genes (Fig. 2a), indicated potential novel molecular links to ALS. To explore shared pathways within these overlaps, we identified two common gene modules across the datasets, which were related to gene ontology terms ‘proteins localised in the ER’ (Fig. 2b, c) and ‘Golgi-vesicle transport’ (Supplementary Fig. 2a-c). Those genes in these modules that showed corresponding changes at transcriptional and protein levels were then selected as top candidates with a high potential to influence astrocyte-specific ALS pathways. Amongst these *KIF5A, HLA-DPA, RPS4Y1* had low expression and *DYNC1I1*, *LMAN1* showed high expression in human SOD1 ALS astrocytes (Fig. 2d). When mapped on PPI-networks, proteins coded by four of the five top candidate genes were found to interact with proteins involved in intracellular transport (Fig. 2e). While KIF5A dysfunction has been implicated in axonal transport disturbances in ALS^21,22^, its expression and function have been unexplored in astrocytes. Therefore, in our subsequent analyses, we specifically focussed on the role of KIF5A in astrocytes.

### Low KIF5A levels result in reduced astrocyte polarity and shorter processes

**Fig. 3:**
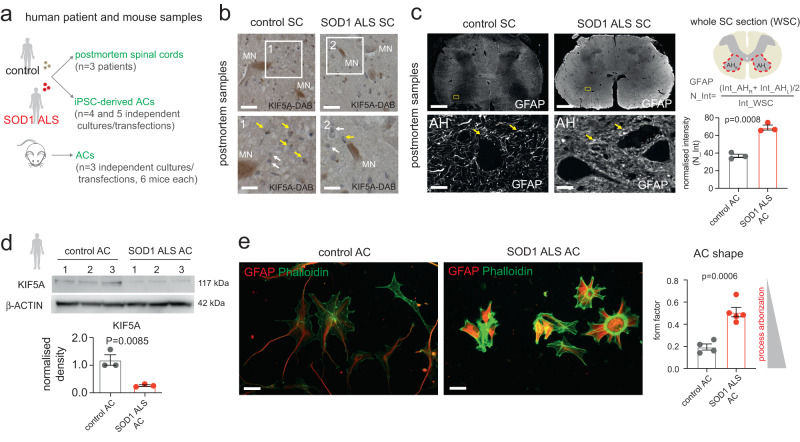
Detection of low KIF5A immunoreactivity and shape changes in SOD1 ALS astrocytes. **a** Schematic illustration of human and mouse experimental platforms. **b** Images demonstrating KIF5A immunolabelling with DAB intensification and nuclear counterstain in control and SOD1 ALS human postmortem spinal cord sections. Large cells with motor neuron morphology (MN) display KIF5A immunoreactivity, while cells with smaller nuclei either have weak IR (yellow arrows) or no IR (white arrows). Insets represent magnified areas (also see Supp. Figure 3a). **c** Representative immunofluorescence images (left) of glial fibrillary acidic protein (GFAP) immunoreactivity in human ALS post-mortem spinal cord sections and controls. Arrows indicate GFAP+ astrocyte (AC) processes in insets. Schematic diagram (middle) summarising the normalisation of GFAP signal intensity measurements (mean grey values) across immuno-stained histological sections. Graph (right) represents the normalised intensity values in each group. *n* = 3 ALS and 3 non-ALS control patient samples (3 spinal cord sections per patient). **d** Western blot (WB) images of KIF5A immunoreactive bands in SOD1 ALS and control iPSC-derived astrocyte (AC) samples. Graph shows band densities normalised to control (1) and to b-actin bands. *n* = 3 batches for ALS and control AC cultures. **e** Representative immunofluorescence images of human control and ALS GFAP+ ACs co-stained with phalloidin (for f-actin) and quantification of their shape, defined by the form factor. *n* = 4 independent control and 5 ALS AC cultures. For each analysis, data represent mean ± SEM with unpaired two-tailed t-tests. Scale bars: 20μm for b, 1 mm for c (50μm for insets), 40μm for e. Please, also see Suppl. Figure 3, Suppl. Figure 7.

**Fig. 4:**
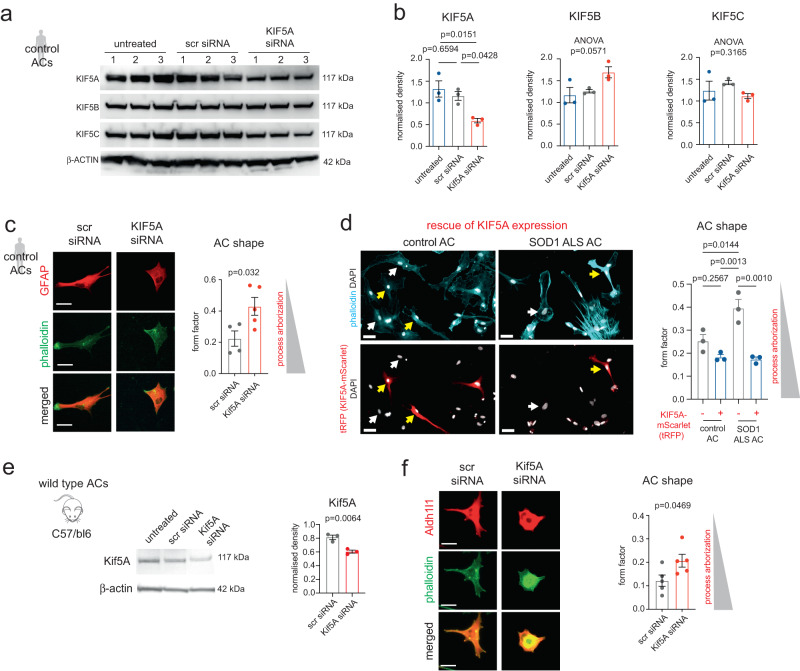
Inhibition of KIF5A expression in astrocytes recapitulates SOD1 ALS astrocyte morphology. **a** WB images of KIF5A, KIF5B and KIF5C immunoreactive bands for untreated, scr siRNA-treated and KIF5A siRNA-treated healthy human control astrocyte (AC) samples. **b** Graphs show band densities normalised to β-ACTIN bands. *n* = 3 independent AC culture batches and transfections. **c** Representative immunofluorescence images of human control and ALS GFAP+ ACs co-stained with phalloidin (for f-actin) and quantification of their shape, defined by the form factor. *n* = 4 independent control and 5 ALS AC cultures. Data represent mean ± SEM with unpaired two-tailed t-test. **d** Representative immunofluorescence images of control and SOD1 ALS ACs transfected with the KIF5A-mScarlet expression plasmid, showing KIF5A-mScarlet+ ACs (red, tRFP immunoreactivity) and non-transfected KIF5A-mScarlet- ACs (non-red) with F-ACTIN labelling by phalloidin-488 (cyan) and DAPI-stained nuclei (white). Graph represent the quantification of AC shape, defined by the form factor. *n* = 3 transfected cultures in each condition. Data represent mean ± SEM with one-way ANOVA with Tukey’s posthoc test. **e**, **f** Equivalent analysis and illustrations to a-c for wild-type C57BL/6 mouse ACs. For e, *n* = 3 independently transfected AC cultures (6 mice per culture); for f, *n* = 5 independently transfected cultures per group. Data represent mean ± SEM with unpaired two-tailed t-test. Scale bars: 50μm for c and f, 30μm for d. Please, also see Suppl. Figure 4, Suppl. Fig. 8.

We examined whether the KIF5A-related disturbances indicated by our integrative multi-omics analysis result in reduced KIF5A protein levels in astrocytes and alterations in their morphology. This objective was supported by a study implicating KIF5A in microtubule maintenance in polarised cells^23^. First, we immunostained human SOD1 ALS and control patient-derived postmortem spinal cord tissue sections for KIF5A and glial fibrillary acidic protein (GFAP) (Fig. 3a–c). Immunohistochemistry in the human spinal cord tissue detected no or only low levels of KIF5A in cells other than large motor neurons in the spinal cord, similar to that found in other studies^18^ (Fig. 3b; Supplementary Fig. 3a**;** Supplementary Table 1). Although, we demonstrated that KIF5A immunoractivity (IR) was associated with GFAP+ processes in some astrocytes in the postmortem tissue (Supplementary Fig. 3a), this method was unsuitable for precisely detecting the KIF5A content in functionally important fine astrocytic processes that are GFAP negative^13,24^. These findings reduced the feasibility of establishing a correlation between the astrocyte process KIF5A abundance and cytoskeletal alterations in the SOD1 ALS samples. Astrocytic cytoskeletal changes were indicated by a 1.88-fold increase in GFAP IR in ALS versus control spinal cords (Fig. 3c). Thus, to increase the detection yield for KIF5A IR and to allow mechanistic observations, we used human (Supplementary Fig. 3b) and mouse astrocyte cultures following the confirmation of KIF5A antibody specificity (Supplementary Fig. 4a–c, Supplementary Table 1). Using immunoblots, we verified KIF5A protein levels in astrocyte cultures derived from either a human SOD1 ALS or a control iPSC line corresponding with the experimental subjects in the aforementioned multi-omics analyses^14^. This confirmed KIF5A protein content in control astrocytes and a significantly lower level in ALS astrocytes (Fig. 3d). Next, we used the dynamic astrocyte culture platform for mechanistic investigations into the role of KIF5A. For this, we used phalloidin, an F-actin stain, to visualise cytoarchitecture and potential shape changes. It helped define the form factor (FF), the ratio of cell perimeter length to area measurements, a sensitive marker of cell shape. High FF values (closer to 1) define rounder cells with shorter processes, and low values (closer to 0) represent cells with longer processes in astrocytes identified by GFAP immunolabelling (Fig. 3e, Supplementary Fig. 4c). At 30 DIV, ALS astrocytes displayed shorter processes, representing a 2.62-fold greater FF value when compared to their healthy control counterparts (Fig. 3e), raising the possibility of the involvement of KIF5A in astrocytic polarity. To elucidate the potential causative relationship between low KIF5A levels and cytoskeletal/process alterations, we utilised a siRNA-based knock-down (KD) strategy in control patient-derived human astrocytes. First, we confirmed the KD effect in KIF5A protein amounts versus their scrambled (scr) siRNA-treated counterparts (Fig. 4a,b), while the levels of KIF5B and KIF5C subunits remained unchanged. Then, human astrocytes identified by GFAP/phalloidin staining were subjected to cell shape analysis. FF values were increased by 1.92-fold for KIF5A siRNA-treated human astrocytes compared to their scr siRNA-treated counterparts, indicating process shortening or reduced arborisation when KIF5A protein levels were diminished (Fig. 4c). Then, to verify that KIF5A deficiency in human SOD1 ALS astrocytes is directly responsible for the less arborized phenotype, we transfected these cells with a KIF5A-mScarlet expression plasmid and assessed their morphology against non-transfected cells. This rescued the phenotype of SOD1 ALS astrocytes, as indicated by the significantly lower FF values, while it did not induce further process complexity in non-mutant control astrocytes (Fig. 4d). The KD effect on KIF5A protein levels and cytoskeletal changes were also recapitulated in primary cultures of astrocytes derived from wild-type C57BL/6 mice (Fig. 4e, f). These findings revealed the conserved presence and the regulatory role of KIF5A in human and mouse astrocytes, impacting process morphology.

### KIF5A knockdown recapitulates the reduced KIF5A distribution, mitochondrial traffic and microtubule organisation observed in SOD1 ALS astrocyte processes

**Fig. 5:**
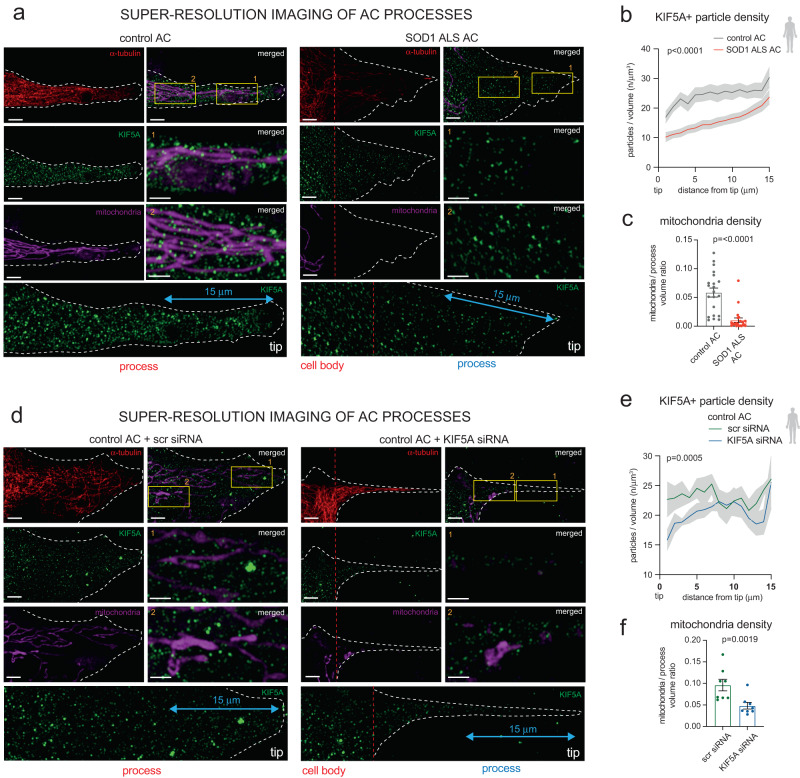
SR-SIM analysis reveals reduced KIF5A and mitochondria density in the processes of SOD1 ALS astrocytes and KIF5A siRNA-treated control astrocytes. **a** Representative SR-SIM immunofluorescence images of control and ALS AC processes, demonstrating α-tubulin-, KIF5A-immunoractivity (IR) and mitoB-stained mitochondria. **b** Plots representing KIF5A+ particle densities within 1μm segments along the distal 15 μm length of the process in control and ALS astrocytes (ACs). n = 22 control, 21 SOD1 ALS AC processes; data is expressed as mean ± SEM (grey bands); unpaired two-tailed t-test. **c** Quantification of mitochondrial density (ratio of mitochondria and process volume) per AC process. *n* = 21 control and 22 SOD1 ALS AC processes; data is expressed as mean ± SD; two-tailed Mann Whitney test. **d** Representative SR-SIM immunofluorescence images of control scr siRNA-treated and KIF5A siRNA-treated AC processes, demonstrating α-tubulin-, KIF5A-IR and mitoB-stained mitochondria. **e** Plots representing KIF5A+ particle densities within 1μm segments along the distal 15μm length of the process in control and ALS ACs. *n* = 7 control+scr siRNA-treated and 7 SOD1 ALS + KIF5A siRNA-treated AC processes; data expressed as mean ± SEM (grey bands); unpaired two-tailed t-test. **f** Quantification of mitochondrial density per AC process. *n* = 8 processes of scr siRNA-treated and KIF5A siRNA-treated AC processes; data represents mean ± SD; two-tailed Mann Whitney test. Scale bars: 4μm (2μm for insets). Please, also see Suppl. Figure 5.

Next, we addressed whether low KIF5A levels can influence kinesin-1/KIF5A-dependent traffic along astrocyte processes and whether this has direct consequences for microtubule (MT) arrangements. This objective was supported by the reported effects of kinesin or KIF5A on MT assembly and stability^23,25^. To do so, we analysed KIF5A distribution in processes in ALS astrocytes and in control astrocytes subjected to KIF5A KD. This study was assisted by super-resolution structural illumination microscopy (SR-SIM)-based particle analysis approach, a low-throughput but highly suitable method for low abundance protein visualisation (Fig. 5a–f). This work revealed that KIF5A immunoreactive particle density was significantly reduced in the distal 15 μm segment of α-tubulin+ ALS astrocyte process tips when compared to controls (Fig. 5a, b). These findings were associated with a reduced presence of mitochondria, a KIF5A cargo^26^, which was established by expressing the ratio of MitoBright (MitoB)-labelled mitochondria and process volumes in control and ALS astrocytes (Fig. 5c, Supplementary Fig. 5a). To assess the dynamics of this process, we performed live imaging of MitoB+ mitochondria and measured their velocity using the ImageJ TrackMate plugin. This demonstrated a 1.57-fold reduction in mitochondrial velocity calculated from the average values for ALS versus control astrocytes, reflecting a reduction in transport efficacy (Supplementary Fig. 5b–d). The reduced KIF5A+ particle and mitochondrial densities seen in ALS astrocytes were faithfully mimicked by the KIF5A KD studies in control astrocytes (Fig. 5d–f). Next, we used SR-SIM to examine the effects of low KIF5A abundance on process microarchitecture. Astrocytes were subjected to α-tubulin immunolabelling, followed by directionality analysis (ImageJ v2.0 plugin) of microtubules in a 30 μm segment measured from the tip of GFAP+ processes. The significantly higher dispersion factor values indicated MT disorganisation in human ALS astrocytes when compared to controls, which was mimicked by KIF5A KD in control mouse astrocytes (Supplementary Fig. 5e, f). Altogether, these findings imply that KIF5A plays a central role in process formation or maintenance and implies its involvement in ALS.

### The overexpression of kinesin-1 regulator JNK1 rescues polarity and KIF5A density in SOD1 ALS astrocyte processes

**Fig. 6:**
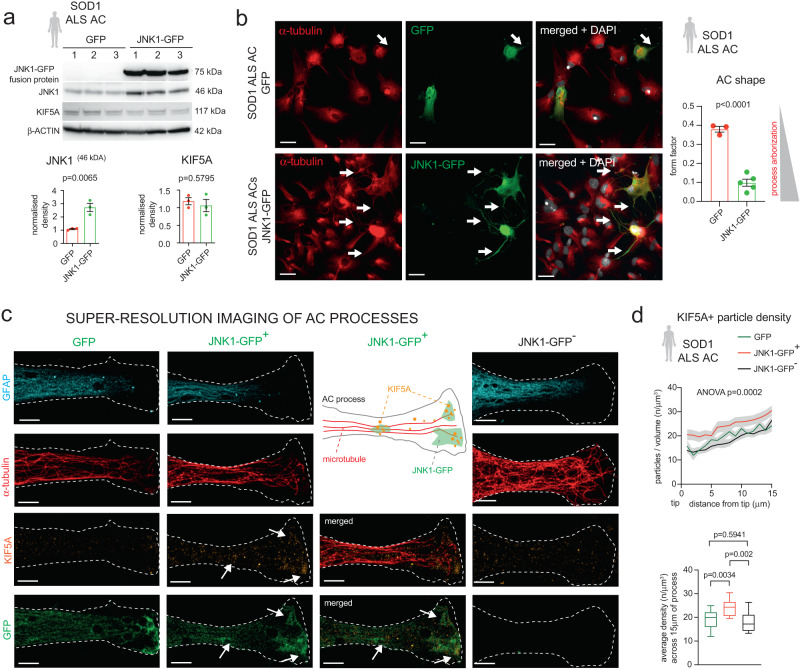
JNK1-overexpression rescues impaired polarity and KIF5A distribution in SOD1 ALS AC processes. **a** Western blot (WB) images of JNK1, KIF5A and β-actin immunoreactive bands in GFP and JNK1-GFP vector transfected SOD1 ALS AC samples. Graphs show band densities normalised to GFP (non-JNK1 + ) AC bands (1) and to β-ACTIN bands. *n* = 3 independent batches of AC cultures and transfections; two-tailed unpaired t-test; data represent mean ± SEM. **b** Representative immunofluorescence images of α-tubulin+ ACs that were not transfected or transfected by JNK1-GFP in SOD1 ALS AC cultures. Graph represents the quantification of AC shape, defined by the form factor. *n* = 3 independently transfected batches of AC for each group; two-tailed unpaired t-test; data represent mean ± SEM. **c** Representative SR-SIM images of GFP + , JNK1-GFP+ and JNK1-GFP- SOD1 ALS AC processes, demonstrating GFAP-, α-tubulin-, KIF5A-IR and GFP-fluorescence. Schematic illustrates the distribution of KIF5A-JNK1-GFP colocalization. **d** Plots (upper graph) representing KIF5A+ particle densities and boxplots (lower graph) showing the average densities in 1μm segments along the distal 15μm length of the process in GFP + , JNK1-GFP- and JNK1-GFP + ALS ACs. *n* = 6 GFP, 18 JNK1-GFP+ and 8 JNK1-GFP- AC processes from two experiments; one way ANOVA and Tukey’s posthoc test; for the upper graph, data represent mean ± SEM (grey bands); for the lower graph, line, box and whiskers represent median, upper and lower quartiles and 5-95 percentiles, respectively. Scale bars: 40μm for b, 2μm for c. Please, also see Suppl. Figure 6, Suppl. Fig. 9.

To examine if low KIF5A expression observed for ALS astrocytes is responsible for its process phenotype, we overexpressed Jun N-terminal kinase-1 (JNK1), a kinesin regulator, by transfecting astrocytes with a JNK1-GFP fusion protein-expressing plasmid. We chose this strategy as the interaction between JNK1 and kinesin-1 is known to promote microtubule growth^23^. Moreover, from a practical perspective, kinases are pharmacologically more targetable than motor proteins or their subunits, such as KIF5A^27,28^. First, we confirmed JNK1 protein expression in SOD1 ALS astrocytes transfected by either the JNK1-GFP or the control GFP plasmid, using immunoblots and fluorescence microscopy. The western blots showed a 2.51-fold increase in JNK1 protein amounts (46 kDa) and exclusive JNK1-GFP fusion protein (75kDA) abundance in JNK1-GFP transfected astrocytes, demonstrated by either JNK1 (Fig. 6a) or GFP immunolabelling (Supplementary Fig. 6a) and GFP-fluorescence in astrocytes (Fig. 6b), while the KIF5A protein levels remained equally low (Fig. 6a). We then compared the shapes of GFP- and JNK1-GFP transfected astrocytes detected by GFP fluorescence. JNK1 overexpression dramatically induced process formation in SOD1 ALS astrocytes, which was marked by a 3.85-fold decrease in FF (Fig. 6b). Next, to address whether improvements in kinesin-1/KIF5A distribution could underlie JNK1 overexpression-induced process formation, we performed SR-SIM analysis of KIF5A/GFP co-labelling in GFP-transfected (GFP + ) SOD1 ALS astrocytes and in JNK-GFP-expressing (JNK-GFP+) and non-JNK-GFP-expressing cells (JNK-GFP-) in JNK-GFP-transfected ALS astrocyte cultures. KIF5A and JNK1-GFP IR showed a similar distribution and co-localisation in the process tips (Fig. 6c). Overall, KIF5A particle density was significantly greater along the distal 15 μm segment of α-tubulin+ process tips in the JNK1-GFP-tranduced group compared to GFP+ or JNK1-GFP- processes (Fig. 6d). Since KIF5A protein levels remained unaltered, this indicated that JNK1 promotes kinesin-1/KIF5A motility and recruitment into processes of ALS astrocytes.

### JNK1 overexpression restores mitochondrial abundance for EAAT2 association in SOD1 ALS astrocyte processes

**Fig. 7:**
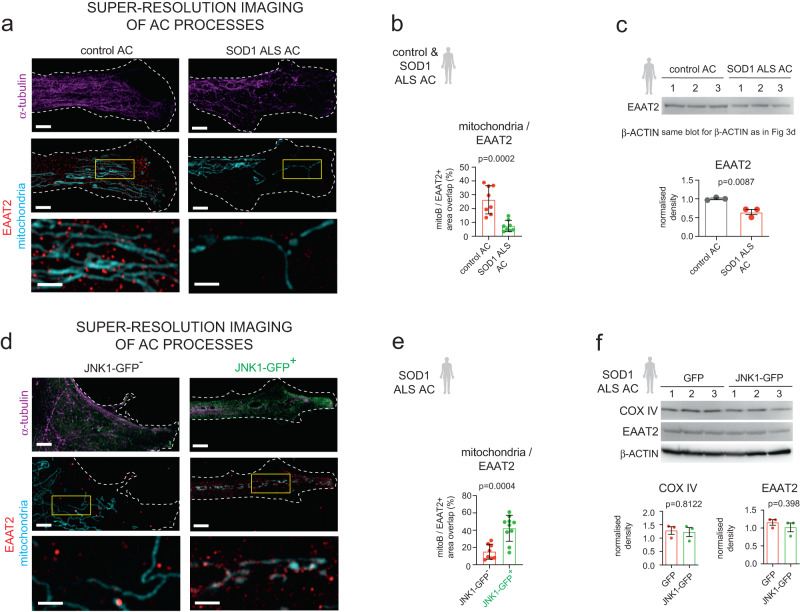
JNK1-overexpression improves mitochondrial abundance for EAAT2 association. **a** Representative SR-SIM immunofluorescence images of control and ALS ACs, demon- strating α-tubulin-, EAAT2-IR and mitoB-stained mitochondria. Insets (lower image panels) illustrate magnified areas indicated by the yellow boxes. **b** Plots illustrate the percentages of mitoB + /EAAT2+ area-over- laps in control and SOD1 ALS AC processes. *n* = 8 control and 8 SOD1 ALS ACs (5-6 images per AC from two experiments); Data represents mean ± SD. **c** Western blot (WB) images of EAAT2 immunoreactive bands in control and SOD1 ALS AC samples. Graph shows band densities normalised to control (1) and to β-actin bands. n = 3 independent batches of control and ALS AC cultures; data represent mean ± SEM. **d** Representative SR-SIM immunofluorescence images of non-or JNK1-GFP-transfected ALS AC processes, demonstrating a-tubulin + , EAAT2+ and mitoB-stained (mitochondria) objects. Insets (lower image panels) illustrate magnified areas indicated by the yellow boxes. **e** Graph illustrates area-overlaps between mitoB + /EAAT2+ immunoreactive objects in AC processes. *n* = 8 JNK1-GFP- and 10 JNK1-GFP+ ACs from two experiments (5-6 images per AC from two experiments); data expressed as mean ± SD. **f** WB images of COX IV and EAAT2 immunoreactive bands in GFP and JNK1-GFP vector transfected ALS AC samples. Graphs show band densities normalised to GFP (non-JNK1 + ) AC bands (1) and to b-actin bands. *n* = 3 independently transfected AC culture batches per group; data expressed as mean ± SEM. For each analysis, unpaired two-tailed t-test was used. Scale bars: Scale bars: 2μm (1μm for insets). Please, also see Suppl. Fig. 10.

Next, we addressed whether JNK1 overexpression-induced increase in KIF5A process density has a consequence for mitochondrial availability^26^ and potential coupling with the human glutamate transporter EAAT2 in ALS astrocyte processes. This association emerges as an important energy-providing step for astrocyte process plasticity^29^, which helps regulate neuronal homoeostasis. For this, we compared the proportion of MitoB-labelled mitochondrial areas overlapping with EAAT2 IR in control and ALS astrocyte processes, which showed a 3.56-fold lower EAAT2/MitoB area-overlap in the latter (Fig. 7a,b). To examine if this could be attributed to the loss of mitochondrial cargo rather than to lower EAAT2 levels that characterise astrocytes in ALS^30,31^, we measured relative EAAT2 levels compared to controls. This analysis showed only a 35.18% reduction in EAAT2 protein amounts in ALS astrocytes (Fig. 7c). Overall, the more subtle alterations in EAAT2 levels relative to the greater changes in EAAT2/mitochondrial area-overlaps suggest that the KIF5A-dependent transport failure may also contribute to EAAT2 dysfunction in ALS.

Finally, to address whether the deficiency of mitochondrial transport may be a result of impaired kinesin-1/KIF5A distribution in SOD1 ALS processes, we repeated the measurements in astrocytes expressing JNK1-GFP (Fig. 7d). We found a 2.79-fold increase in the proportion of EAAT2/MitoB area overlaps in processes of JNK1-GFP-expressing ALS astrocytes versus their non-transfected GFP-counterparts (Fig. 7e). Having established by western blotting that overall neither mitochondrial cyclo-oxygenase (COX) IV nor EAAT2 protein levels are altered upon the transfection by the JNK1-GFP plasmid (Fig. 7f), our data suggest that JNK1-overexpression improves the transport of mitochondria into the processes and their potential association with EAAT2. Overall, our findings indicate that despite the low KIF5A levels, process structure and mitochondrial availability can be rescued by JNK1 in distal parts of SOD1 ALS astrocyte processes. These results corroborate the likely involvement of KIF5A-associated traffic in astrocyte-related ALS pathogenesis.

## Discussion

Here, we demonstrate the convergence of ALS pathways onto key processes related to dysregulated cellular transport involving KIF5A, a kinesin-1 heavy-chain subunit that has not been visualised in astrocytes previously. We show direct experimental evidence that KIF5A is widely distributed along astrocyte processes and its deficiency leads to disruption of structural integrity and mitochondrial transport, suggesting a loss of energy supply to distal process segments. This paradigm also implicates an underlying cause for cytoskeletal alterations in SOD1 ALS astrocytes characterised by impaired kinesin-1/KIF5A distribution as JNK1, a kinesin regulator, can restore the phenotype.

Our multi-dimensional discovery pipeline pinpoints a key gene expression and pathway signature in ALS, implying a common pathogenic route. Previous efforts to integrate genetic associations and gene expression have primarily focused on expression quantitative trait loci (eQTL) analysis where single nucleotide polymorphisms (SNPs) linked to a disease via GWAS are also shown to be linked to changes in mRNA/protein levels^32^. A recent study also explored ALS pathway signatures based on polygenic score analysis, which were found to be enriched not only in neurons but also in glial cells^5^. However, combining these methods with biological validations in various cell populations, including non-neuronal cells, is a prerequisite for addressing the pathogenicity of genetic variants and their key effectors. This is highly relevant, given the limited availability of robust brain cell type-specific eQTL databases. The integrative approach shown in our work illustrates how network-expansion analyses can be exploited to integrate cell type-specific differential expression data with genetic evidence despite minimal overlap at the gene level, while discovering important pathological cellular pathways.

We have identified five genes mapped within a protein interaction module that shows enrichment in ALS-related gene-network expansion elements and in astrocytic RNA/protein changes. Ontologies for four of these genes implicate involvement in intracellular transport. Amongst these, we focused on KIF5A for an in-depth study for multiple reasons: their presence and function have not been previously described in astrocytes; neuronal kinesin-1/KIF5A dysfunction has been implicated in a broad range of diseases caused by KIF5A-mutations, including ALS^15,16^, Charcot-Marie-Tooth type 2 disease (CMT2) and hereditary spastic paraplegia (HSP)^33,34^; kinesin1/KIF5A-related perturbations can also occur in non-KIF5A mutation-related ALS forms^21,22^. Our integrated network-expansion study supports that kinesin-1/KIF5A-related pathway disturbances are more broadly shared and that mechanisms could vary across different ALS genotypes, including gain of toxic function or loss of function. For instance, KIF5A(Δexon27) mutant oligomers are hyperkinetic, aggregate, dysregulate transport^35–37^ and cause similar SOD1 protein aggregations seen in the SOD1^D90A^ mutation^38^, leading to axonal cytoskeletal changes and cytotoxicity. In contrast, our SOD1 ALS mutation-related paradigm demonstrates loss of KIF5A function through decreased expression and protein levels. Due to the close functional coupling of astrocyte processes and synapses^39^, kinesin-1/KIF5A-related astrocytic abnormalities could worsen the breakdown of neuronal networks in ALS.

Interestingly, a genetic variation within another molecular motor, the kinesin-associated protein 3 gene was proposed as a disease-modifying factor associated with increased survival or with an upper MN-predominant phenotype in sporadic ALS^40,41^. However, no link was found in broader population-based studies^42,43^. This suggests complexity in human genetic studies evaluating genetic risks and influences, highlighting the importance of alternative integrative frameworks supported by validations in biological platforms.

In contrast to previous observations^18^, we show that KIF5A is expressed in astrocytes, albeit at low levels. The demonstration of astrocytic KIF5A content in CNS tissues and its alterations in ALS may have been hampered by difficulties in detecting low protein levels by immunohistochemical approaches. Although the use of SR-SIM is confined to low-throughput analyses, our mechanistic studies using this method allowed us to detect and track KIF5A+ particles despite ultra-low protein abundance in processes of cultured control and SOD1 ALS astrocytes. SR-SIM, along with morphological analyses, were also central to showing that low KIF5A levels lead to disturbances in MT architecture and process formation. It is likely to underlie the SOD1 ALS astrocyte phenotype as restored KIF5A expression rescues their morphology. This conclusion is supported by studies on neurons, demonstrating kinesin-dependent MT elongation^44^, organisation^45^ and stability^25^, which influences cargo distribution in polarised cells.

The dramatic effect of KIF5A KD on astrocyte morphology, with unchanged protein levels of KIF5B and KIF5C subunits, suggests that KIF5A has a non-redundant function in certain cell types. It is supported by studies showing that KIF5A mutation-related dysfunction remains uncompensated in diseases and its unique ability to affect mitochondrial localisation, which is essential in process maintenance^26^.

Aligned with the observed KIF5A-dependent MT and cytoarchitectural abnormalities, we found a decline in mitochondrial traffic and distribution in SOD1 ALS astrocyte processes. Our results suggest that this can be attributed to the diminished availability of KIF5A for anchoring mitochondria, leading to kinesin-1-related traffic impairment. In addition to the low KIF5A abundance, other plausible mechanisms may have also facilitated mitochondrial cargo issues in SOD1 astrocytes. For instance, the mutant SOD1 protein has been demonstrated to reduce mitochondrial transport in transfected cortical neurons by lowering Miro1 levels, a protein anchoring KIF5A^21^, and also potentially through P38 MAP kinase-dependent phosphorylation of kinesin-1^46^. Similar decoupling were also observed in C9ORF72^22^, VAPBP56S^47^, and FUS^48^ ALS mutations, which broadens the relevance of KIF5A in ALS pathogenesis.

Our results, together with the above findings, highlight that targeting KIF5A could potentially improve kinesin-1-mediated cargo in astrocytes and neurons in ALS. Since we found that human astrocytes have low baseline KIF5A levels, it is plausible to speculate that even a small improvement in KIF5A protein levels or activity would amend astrocyte process morphology and function. Previous work indicated that JNK1, a kinesin-1/KIF5 transport regulator^49^, stimulates MT elongation in HeLa cells when MT arrangements are disrupted without affecting kinesin-1 levels^23^. Our study corroborated this observation and provided direct evidence that process-formation and mitochondrial transport can be improved in human SOD1 ALS astrocyte process tips by JNK1 overexpression. It seems to compensate for the loss of KIF5A availability, functionally. While more investigations are required, this suggests that JNK1 can enhance kinesin-1 motility and transport function in SOD1 ALS astrocytes, similarly to that seen for rodent neurons^49^. Although it remains to be explored whether pharmacological activation of JNK1 is a plausible approach – especially in light of emerging small-molecule kinase activators^28^ – our findings demonstrate a proof-of-principle strategy for restoring astrocyte process structure and cargo distribution in ALS.

In our paradigm, astrocyte process and cargo disturbances represent a loss of function (LOF) phenotype, supporting the emerging LOF effects^12^ in ALS, in addition to the well-described gain of toxic functions^11^. This phenotype may cause neuronal network dysfunction by reducing synapse coverage and support by impaired astrocyte process arborisation or perturbations to glutamate clearance. In particular, our findings implying diminished mitochondrial association with EAAT2 due to low astrocytic KIF5A levels suggest a mechanism underlying glutamate transport dysfunction^50^, in addition to downregulated EAAT2 levels, one of the earliest observed astrocyte-related molecular pathologies in ALS^31,51^. However, more functional assessments are required to explore this possibility further. In contrast to LOF, an integrative proteomics-based regulatory network study by Mishra and colleagues^52^ have recently found that ligand-receptor interactions mediate a toxic gain of SOD1 ALS astrocyte function through releasing APP that exerts MN toxicity by DC6-signalling. Both experimental examples highlight the need for advanced system biology approaches with the additional integration of human and mouse multi-omics studies to explore the full picture of loss or gain of function pathways in astrocytes, potentially guiding a multi-target strategy in ALS.

We propose that our pipeline is a powerful tool for identifying cell type-specific biological pathways, which led us to discover the presence of KIF5A in astrocytes and its regulatory role for process integrity and cargo regulation. Our work also indicates that modest improvements in kinesin-1/KIF5A-dependent transport may improve astrocyte process-mediated support of neuronal networks with relevance to ALS therapeutics.

## Methods

### Human interactome and network-based expansion

For the human interactome, we selected STRING v11.0 (combined score > 0.75), BioGRID (v 3.5.172) and IntAct (May 2019) databases derived from high-throughput studies (reporting at least 1,000 interactions). All nodes were mapped to Ensembl gene (ENSG) identifiers, edges were considered as non-directed connections and self-loops and duplicated edges were eliminated from the interactome. For the network-based expansion approach, the input hits were mapped to the interactome and their initial weights were diffused using Personalised PageRank (PPR) algorithm from the igraph R package (https://igraph.org/r/). The nodes receiving the 25% upper part of the PageRank ranking score were selected for community detection, using walktrap clustering in igraph. Resulting communities with more than 300 nodes were subjected to further rounds of clustering until threshold values were reached. Communities with >10 nodes were defined as modulesand as significant modules if their PageRank score was calculated as significant (adjusted *p* value < 0.05) by Kolmogorov Smirnov test followed by Benjamini-Hochberg post hoc analysis.

### GWAS Catalogue of neurodegenerative diseases

We selected nine neurodegenerative diseases from the parent term EFO:0005772 for neurodegenerative disease in the Experimental Factor Ontology (EFO) database (https://www.ebi.ac.uk/efo/), which had at least two genes mapped to significant SNPs in the GWAS Catalogue (https://www.ebi.ac.uk/gwas/). The -log10 (*p* value) was used as starting weight for network expansions, selecting the highest value when redundancies were present. The ALS (EFO_0000253, replaced by MONDO_0004976) and sporadic ALS (EFO_0001357) databases were pooled. For bulk Jaccard index calculations (Fig. 1c), all genes found in significant modules were selected for each disease. The same principles were applied to the analysis in Fig. 1d, but only pairs with a ≥ 0.7 Jaccard index were chosen for further analyses, resulting in 10 groups of overlapping modules. For each group of overlapping modules, Gene Ontology Biological Process (GOBP) enrichment (Fisher test with Benjamini-Hochberg post hoc analysis) was expressed as moduler identifier.

### Multi-omics data integration

For multidimensional data integration, the filtered GWAS dataset was combined with astrocyte-related transcriptomic and proteomic datasets that were extracted from published studies^14^ and databases (accession code: GSE102903). The proteomics dataset was further analysed using the Perseus software (https://maxquant.net/perseus/), and the significance A value was calculated from averaged ratios. For network expansion, the -log10 of the reported *p* values was used as a starting signal for each set. Module overlap significance was defined by the Jaccard index. In addition, to further characterise the triple overlaps (GWAS/transcriptomics/proteomics), the GOBP enrichment analysis was performed using the Fisher test with Benjamini-Hochberg correction.

### PageRank score benchmarking

Three groups were revealed as True Positive (TP) gene-disease relations by the following steps. From the DISEASE database, we extracted all genes linked to three neurodegenerative diseases: ALS, AD and PD. Next, we selected all associations that were greater than the third quantile (25% most significant hit: sco>Q3) and the ones with the maximum score (max), which resulted in two groups. Finally, we extracted drug targets from the ChEMBL database assembled using findings by Phase II and phase IV clinical trials for ALS, AD and PD. Areas under the receiver operating characteristic (ROC) curves were calculated using the pROC R package.

### Human postmortem tissue and iPSC line sources

Details for all human samples are included in Supplementary Table 1. Human samples were obtained and handled according to the UK Human Tissue Authority regulations and local guidelines in the laboratories. The postmortem spinal cord tissue samples were obtained from and processed in the London Neurodegenerative Diseases Brain Bank at King’s College, London by C.T. (HTA licence reference: 12293; human tissue bank ethics reference: 18/WA/0206; consent provided by the Brain Bank). The commercial human iPSC-lines were obtained from the European Bank for Induced pluripotent Stem Cells (EBiSC) and the National Institute of Neurological Disorders and Stroke (NINDS) repository by the A.L. laboratory at the University of Cambridge under material transfer agreements (Ref: RG92224, G107192).

### Human astrocyte differentiation

Astrocyte differentiation was performed using a published protocol^14,53^. In brief, human iPSCs were used to generate spinal neural stem cells (NSCs) which were expanded for ~100 days. Expansion was carried out in N2B27 media containing 10 ng/ml FGF-2, and cells were passaged using Accutase at 70% confluency. Then two subsequent rounds of cell sorting were carried out using GLAST antibody-tagged magnetic beads (Miltenyi Biotec, 130-095-825) to obtain pure glial cell progenitor cultures (GPCs), according to the manufacturer’s protocol. GPCs were frozen and banked before being differentiated into astrocytes by supplementing N2B27 culture media with BMP-4 (Peprotech, 120-05) and hLIF (Peprotech, 300-05), both at 10 ng/ml, for four weeks.

### Mouse astrocyte cultures

Mice were housed and used in accordance with the Home Office Animal Scientific Procedures Act (ASPA), 1986 under project licence P98A03BF9. The upper cervical spinal cord-lower brainstem tissue was dissected from newborn (P0) male and female mice (*n* = 6 mice/each culture; C57BL/6 J, RRID: IMSR_JAX:000664 l; provided by Dr Andrea Loreto). The tissue was physically dissociated into small pieces, followed by enzymatic digestion with 0.25% trypsin and 0.2% collagenase in a mechanical dissociator (Gentle MACS Octo dissociator, Milteny Biotec) at 37 °C for 30 minutes. The obtained single cell suspension was plated in poly-D-Lysine coated T75 flasks and kept in DMEM supplemented with 10% foetal bovine serum (FBS) and 1% antibiotic-antimycotic mix (Thermo Fisher Scientific, 15240096). At day 7, top dwelling progenitor cells and microglia were removed by overnight shaking at 100 rpm; then the culture was differentially passaged using 0.0025% trypsin to remove microglia. Finally, 0.025% trypsin was applied for 2-3 minutes to detach astrocytes for replating, while fibroblast mostly remained attached. Further purification was carried out by GLAST antibody-tagged magnetic beads by published methods^14^, which resulted in high-purity astrocyte cultures ( > 98%).

### Western blots

Cell lysates for protein samples were isolated from astrocyte cultures at 50-60DIV or from HEK293 cells 48 h after transfection, using the RIPA lysis buffer (Sigma-Aldrich, #R0278) supplemented with Pierce Protease Inhibitor Mini Tablets (Thermo Fisher Scientific, #31462) and Pierce Phosphatase Inhibitor Mini Tablets (Thermo Fisher Scientific, #A32957). For astrocytes, 14μg of protein and for HEK293 cells, 20 μg of protein was resolved by SDS-PAGE and then transferred onto PVDF membranes. Then, membranes were incubated overnight with primary antibodies, except for the directly HRP-tagged β-actin antibody (1-hour incubation) used as a loading control. HRP-tagged secondary antibodies were applied for 1 h at room temperature (RT), and the blots were developed using the enhanced chemiluminescence system (GE Healthcare, #RPN2232).

### Immunocytochemistry

Cells on coverslips were washed once with PBS and subsequently fixed with 4% PFA (Thermo Fisher Scientific, 28908) for 12 minutes. Fixed cells were washed with PBS three times, followed by blocking with 0.3% Triton-X (Sigma, T8787) containing 10% normal goat serum (NGS - Sigma, G9023) for 1 hour. Then cells were incubated with the respective primary antibodies in 0.1% Triton-X containing 3% NGS for 2 hours at RT. Secondary antibody incubation was performed for 1 hour at RT. Phalloidin was applied together with the secondary antibodies. For nuclear labelling, cells were incubated with DAPI (Sigma, D9542) in PBS for 10 minutes at RT. Coverslips were mounted on slides using FluorSave (VWR, 345789) for conventional confocal microscopy or Prolong gold (Molecular Probes, P10144) for SR-SIM. The list of antibodies and their details are provided in Supplementary Table 2.

### Immunohistochemistry

For human spinal cord tissue sections, 7 μm paraffin-embedded sections were subjected to deparaffinisation, rehydration and extended microwave antigen retrieval in citrate buffer, followed by blocking in 10% normal goat serum. Primary antibody solutions were added to the slides for 1 hour at 37 °C. After washes in PBS, slides were incubated for 45 minutes at RT with secondary antibodies. The slides were mounted with coverslips using Vectashield with DAPI (Vector Laboratories).

### KIF5A knockdown, KIF5 subunit and JNK1 overexpression

For different downstream applications, human iPSC-derived or mouse primary astrocytes were seeded on geltrex-coated 6-well plates (2 ×10^5^ cells per well) or on coverslips (13 mm diameter, 2 ×10^4^ cells per coverslip) or on confocal 8-well chambers (Ibidi, 80826; 10^4^ cells per well). For *KIF5A* knockdown the lipofectamine RNAiMAX Reagent (Thermo Fisher Scientific, 13778075) was used with 90 nM of KIF5A siRNA or non-targeting siRNA per reaction (for human spinal cord astrocytes: Dharmacon, ON-TARGETplus SMARTpool, L-008559-00-0005; and for mouse cervical spinal cord/brain stem astrocytes: Dharmacon, ON-TARGETplus SMARTpool, L-065418-01-0005). Non-targeting ‘scrambled’ (scr) siRNA was used as control (Dharmacon, ON-TARGETplu Non-targeting Control pool, D-001810-10-05). Cells were analysed 3 days after transfection. For KIF5A or JNK1-overexpression, human astrocytes were seeded on geltrex-coated 6-well plates (2 ×10^5^ cells per well) or on coverslips (13 mm or 17 mm diameter, 2 ×10^4^ cells per coverslip). In 24 hours, astrocytes were transfected with the KIF5A pCMV-mScarlet pSN837 plasmid (Addgene, 184825, a gift from Kyoko Chiba) or in separate experiments, the cells were treated with either a pCMV-JNK1-GFP fusion construct (Addgene plasmid, 86830, gift from Rony Seger) or a pCMV-GFP plasmid (Addgene plasmid, 11153, a gift from Connie Cepko), using Lipofectamine 2000 (Thermofisher Scientific). The cells were analysed three days after transfection, using western blotting, Super-Resolution Structured Illumination Microscopy (SR-SIM; Elyra PS1, Carl Zeiss Ltd), confocal (Leica TCS SPE) or fluorescence microscopy (Leica DM6000). To confirm the KIF5A antibody specificity, HEK293 cells were plated onto T25 flasks and grown in DMEM media supplemented with 10% foetal bovine serum (FBS) until they reached 70-80% confluence and were transfected with either KIF5A (Addgene plasmid pSN837, 184825, a gift from Kyoko Chiba), KIF5B (RC216778, Origene) or KIF5C (RC218796, Origene) plasmids using Lipofectamine 2000 reagent (Thermofisher Scientific). After 48 hours, cells were detached from the flasks using 0.025% trypsin, spun down at 400 g for 5 minutes and processed as lysates by resuspending the pellet in RIPA buffer containing protease and phosphatase inhibitors for immunoblotting.

### Live mitochondrium tracking

Human or mouse primary astrocytes were plated on either geltrex-coated 8-well chambers (Ibidi, 80826) or on CELLview glass-bottom Petri dishes (Greiner Bio One, 627860). Cells were incubated in 500 nM MitoBright Red (a membrane potential independent dye; Dojindo, MT11; gift from Dr András Füredi) diluted in culture media and incubated for 30 min at 37 °C. Media was changed before imaging to Neurobasal without phenol and with 1% Glutamax. Images were taken by a spinning-disc confocal microscope (Olympus IX70 microscope, Hamamatsu ORCA-ER CCD camera, PerkinElmer UltraVIEW scanner controlled by the MetaMorph software) at 1 second intervals. For tracking mitochondrial movements, the TrackMate ImageJ plugin was used.

### Cell shape analysis

The form factor (FF) was then used to compare cell shape/process arborisation affected by decreased expression of KIF5A in SOD1 ALS astrocytes and in siRNA-treated control astrocytes, and in rescue experiments using KIF5A-mScarlet or JNK1-GFP expression plasmids. FF is defined by the following equation: FF = 4π[area]/[perimeter]^2.^, providing a value between 0 and 1. Values closer to 0 represent a polarised cell shape (long and multiple processes), and values closer to 1 indicate circular cell shape (loss of processes). The area and perimeter measurements in ImageJ were based on the visualisation of mScarlet, GFP or F-actin fluorescence by staining astrocytes that were identified by GFAP or ALDH1L1 immunoreactivity and tRFP (Evrogen, AB233), GFP (Proteintech, 50430-2-AP) or phalloidin-488 (Thermo Scientific, A12379) labelling, respectively.

### Super-resolution structured illumination microscopy (SR-SIM) analysis

For SR-SIM, a Zeiss Elyra PS1 (Carl Zeiss GmbH) was used with a 63×1.4NA Zeiss Plan-Apochromat objective, based on a published protocol^54^. Briefly, prior to imaging, fixed and immunostained cells were mounted with 20μl ProLong Gold (P36934, LifeTechnologies) on high-precision 18mm^2^ coverslips (Zeiss Ltd, 170 μm thickness) and allowed to stand at 21-23 °C for 72 hours. The slides were preheated to 23 °C to match the immersion oil temperature in the imaging chamber. Microscopy was performed by the recommended parameters (excitation: 488 nm/200 mW, 560 nm/200 mW and 640 nm/150 mW; structured illumination grid pitches: 28 μm, 34 μm and 34 μm; emission filters: BP495-550, BP570-620,LP655nm). Z-stack through-focus series of images (5-12) were taken at a spacing interval of 0.091 μm in 3 colours sequentially (5 grid phase shifts/rotations). A z-stack with 10 slices covered ~1 μm thickness of the specimen. Image acquisition was performed by a PCO edge sCMOS device (PCO AG, Kelheim, Germany) with a sensor allowing up to 2240×2154 pixel images. The raw data from the multi-dimensional image arrays were processed by the built-in ZEN software. Then, channel alignment was performed, which was based on the calibration data obtained earlier using bead samples, and the output images were saved as.czi files. The microtubule dispersion factor (MDF) was used to compare more subtle changes in the astrocyte process structures between the control and SOD1 ALS group. The measurements for MDF were carried out in astrocyte processes (cytoplasmic protrusions) immunostained for α-tubulin, using the directionalityplugin in ImageJ. Then, the Fourier Components method was applied to build orientation maps in the same plugin. KIF5A immunoreactive particles were visualised by SR-SIM to analyse KIF5A protein distribution and level changes in astrocyte processes. KIF5A+ object segmentation was performed in CellProfiler (v4.2.1; IdentifyObjects) and the distance of each KIF5A+ particle from the process tip was measured using the analyse particle plugin in ImageJ, following equal thresholding across all images. KIF5A+ particle density (n of KIF5A+ particles within each 1 μm segment of process volume across the distal 15 μm length) was quantified in each process. Data were expressed as average for each distance/volume bin (1-15 μm) for comparing particle distribution in distal astrocyte processes between different experimental groups. For mitochondria-EAAT2 immunoreactive area-overlap analysis in double-stained astrocytes, the images were manually thresholded for MitoBright Red fluorescence to accurately identify mitochondria-occupied areas in astrocyte processes. This area was selected in the create selection plugin in ImageJ, and the percentages of overlapping EAAT-2+ territories were measured.

### Image processing and analysis

For immunofluorescence, images were taken either by a fluorescent (Leica DM6000, ×20–63 objectives), confocal (Leica TCS SPE and Nikon Upright Ni-E/A1R) or a super-resolution microscope (for SR-SIM see details above). Camera gain and exposure were kept unchanged while collecting images. For unbiased semi-automated analyses of KIF5A particle and mitochondria distribution or density, unmodified images were uniformly thresholded for the Analyse Particles function or area/volume measurements in ImageJ (v.2.0.0 Fiji) within each experiment. Cell counts were performed manually. Western blot (WB) membrane chemiluminescence was imaged, using the Alliance 4.7 CCD Image System (UVITEC). Quantitative WB analyses were performed in ImageJ, following published standard methods^55^. Band density levels were expressed as fold-changes to controls following normalisation to corresponding β-actin+ bands and to a control sample within the same blot. For micrograph illustrations, the recommended guidelines were followed. Images were minimally processed in ImageJ or Adobe Photoshop (v21.0.3), which was applied equally to samples that were directly compared without compromising data presentation. The processing included uniform changes in gain parameters when clear views were obscured in merged panels. Pseudo-colours in images were rendered in ImageJ for multicolour visualisation in images. For WB illustrations, images were cropped for focussed visualisation, leaving a 6-band width. All uncropped WB images were included in the Supplementary Information. For figure assembly, images were embedded and organised in Adobe Illustrator (v24.0.3). Schematic images of mice were used in the figures, which complies with the content license of pixabay, a royalty-free content-sharing platform.

### Statistics and reproducibility

Statistical test details and exact sample sizes are listed in Supplementary Table 3. Briefly, subject identifiers were blinded for the observers throughout this study. At least three independent biological repeats (independent culture batches/transfections, postmortem patient samples) were used in repeated mechanistic experimental studies, including at least three technical replicates (histological sections, astrocytes). For low-throughput but detailed SR-SIM-based analyses, data acquisition involved multiple astrocyte processes in two or three repeated experiments, and the variance of data across astrocytes or cultures was compared between groups. The GraphPad Prism v.8.0 software was used for statistical analyses and data plotting. When normality was not assumed, nonparametric tests were used. The type of tests, with exact *n* values and *p* values were indicated in the figures and legends with further detail provided in Supplementary Table 3. Statistical significance was accepted at *p* < 0.05.

### Reporting summary

Further information on research design is available in the Nature Portfolio Reporting Summary linked to this article.

## Supplementary information

Supplementary Information

Description of Additional Supplementary Files

Supplementary Data 1

Supplementary Data 2

Supplementary Data 3

Supplementary Data 4

Supplementary Data 5

Supplementary Data 6

Supplementary Data 7

Reporting Summary

## Data Availability

Data that support the results in this work are provided in the Supplementary Data 1-7 files and in the Supplementary Figures 1-11, including the uncropped WB images (Supplementary Information) or are available from the corresponding authors upon reasonable request. The GWAS Catalogue is available at https://www.ebi.ac.uk/gwas/ and the transcriptomic database used is available from GEO (Accession code: GSE102903).

## Code availability

Programme codes are available on Github (github.com/ibarrioh/astrocyte_KIF5A) and in Zenodo (10.5281/zenodo.7681344). Code and software availability is also indicated in the Nature Research Reporting Summary.
