# Supplementary material for: A human proteogenomic-cellular framework identifies KIF5A as a modulator of astrocyte process integrity with relevance to ALS
Source: Commun Biol. 2023 Jun 29;6:678. doi: 10.1038/s42003-023-05041-4 (PMC10310856; doi:10.1038/s42003-023-05041-4)
Supplement: Supplementary file 1 — Supplementary Information [file 42003_2023_5041_MOESM1_ESM.pdf]

ciliary basal body docking (microtubule organisation)

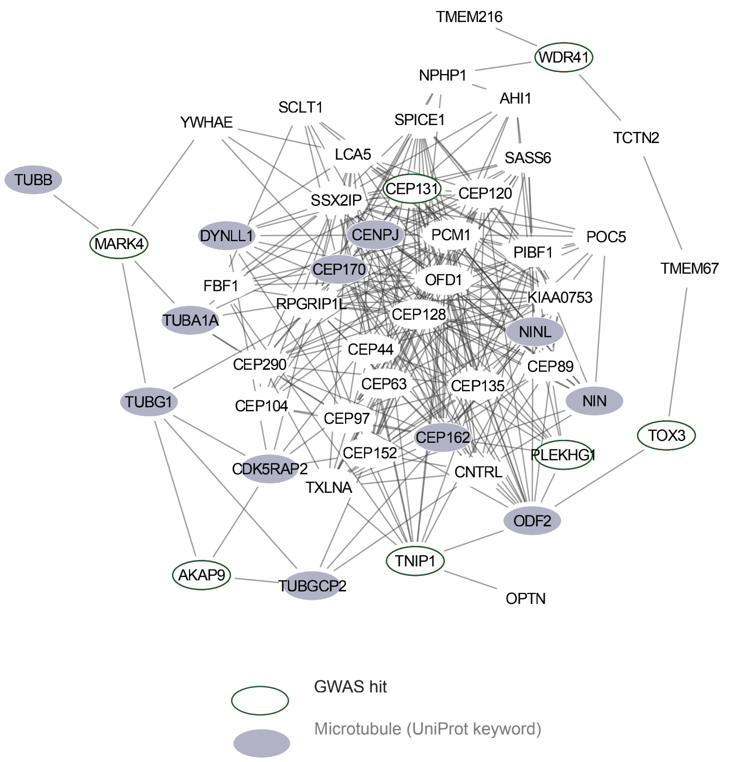

Supplementary Fig 1 Network-based predictions of common pathway disruptions in neurodegenerative diseases. Network of overlapping module 3 representing the GOBP term “ciliary body docking (microtubule organisation)”. Edges represent only the corresponding IntAct protein-protein interactions and their first connections to elements indicating the initial GWAS hits (green node borders). Nodes associated with the Uniprot Keyword term “microtubule” are coded in grey. Supplementary information for Fig. 1d.

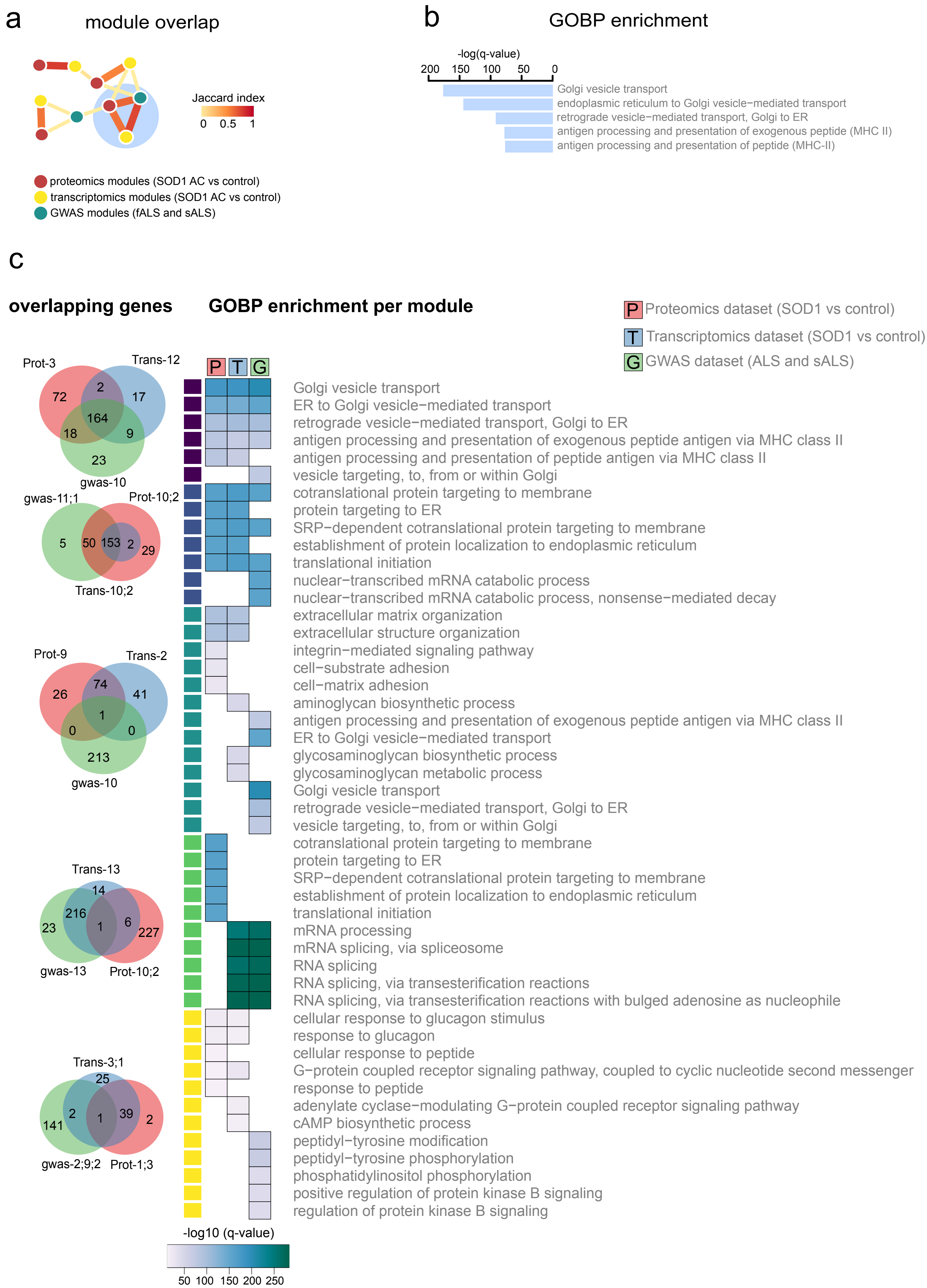

Supplementary Fig 2 Overlaps of significant modules from multi-omics datasets. a Simplified network of overlapping module 1. **b** Corresponding barplots demonstrating the Gene Ontology Biological Process (GOBP) term enrichments (Fisher exact test). **c** Venn diagrams illustrate the number of overlapping elements between the significant proteome, transcriptome and GWAS modules (left panel). Corresponding Gene Ontology Biological Process (GOBP) terms for each overlapping significant module (right panel), in which the colour coding refers to the adjusted p-value (q-value), indicating the enrichment significance (Fisher exact test). Supplementary information for Fig. 2b,c.

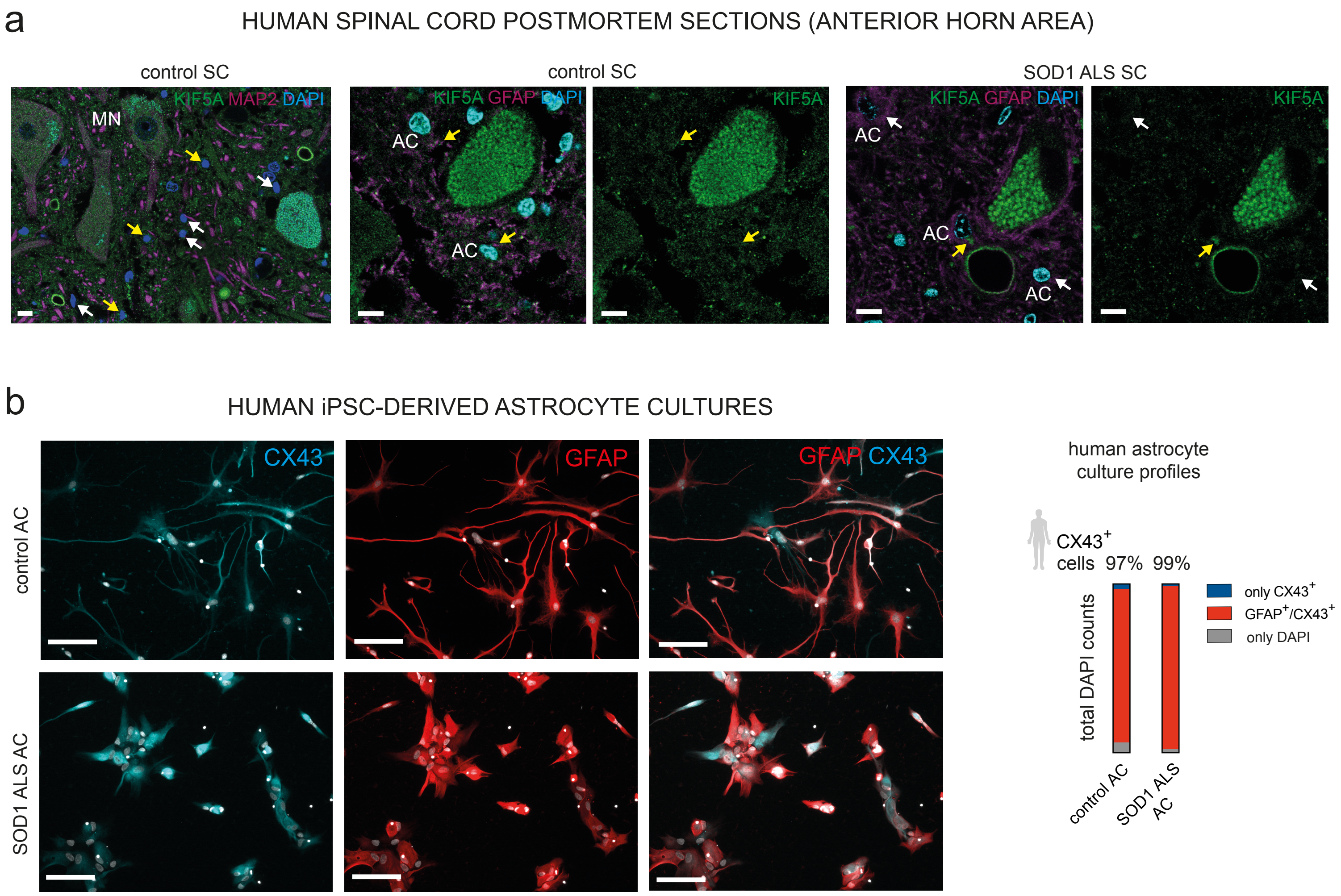

Supplementary Fig 3 Analysis of human control and SOD1 ALS postmortem tissue and astrocyte cultures. a Representative confocal microscopy image (single plane), demonstrating KIF5A (green) and MAP2 (magenta) or GFAP (magenta) immunolabelling with DAPI staining in control human postmortem spinal cord (SC) sections. Large cells with motor neuron morphology (MN) and MAP2+ dendrites display KIF5A immunoreactivity, while MAP2- cells and GFAP+ astrocytes (ACs) with smaller nuclei either have weak KIF5A IR (yellow arrows) or no IR (white arrows), especially in the SOD1 ALS SC samples. **b** Representative fluorescence images (left) of healthy and SOD1 ALS patient-specific induced pluripotent stem cell-(iPSC)-derived ACs (30DIV). Part of the whole charts (right) illustrating the proportion of cells immunoreactive for mature astrocyte (AC) marker, CX43 (>97%) and GFAP in control and ALS AC cultures. Scale bar: 10µm for a, 80µm for b. Supplementary information for Fig. 3a,b,d,e.

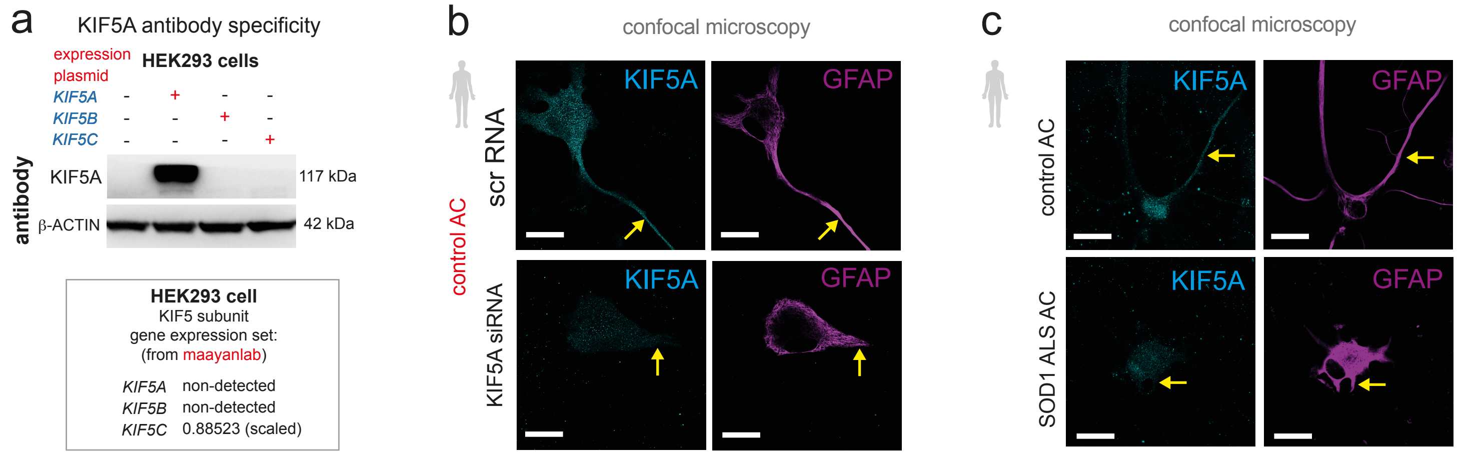

Supplementary Fig 4 KIF5A antibody specificity in HEK293 and human astrocytes. a Representative image of a western blot from protein lysates of HEK293 cells, transfected with either the KIF5A, KIF5B or KIF5C expression plasmid or left non-transfected, demonstrating a band labelled by the KIF5A antibody exclusively in the KIF5A transfected samples (from repeated experiments). Notably, naive HEK293 cells do not express *KIF5A* and show only *KIF5C* expression, according to studies by the mayanlab. **b** and **c** Representative confocal microscopy images, showing speckled KIF5A immunoreactivity in GFAP-immunolabelled processes (arrows) of cultured human control astrocytes (ACs) treated with either scr siRNA or KIF5A siRNA (b) and non-treated control ACs and SOD1 ALS ACs. Scale bar: 20 μm. Please, also see Suppl. Fig. 11. Supplementary information for Fig. 4a,c,f.

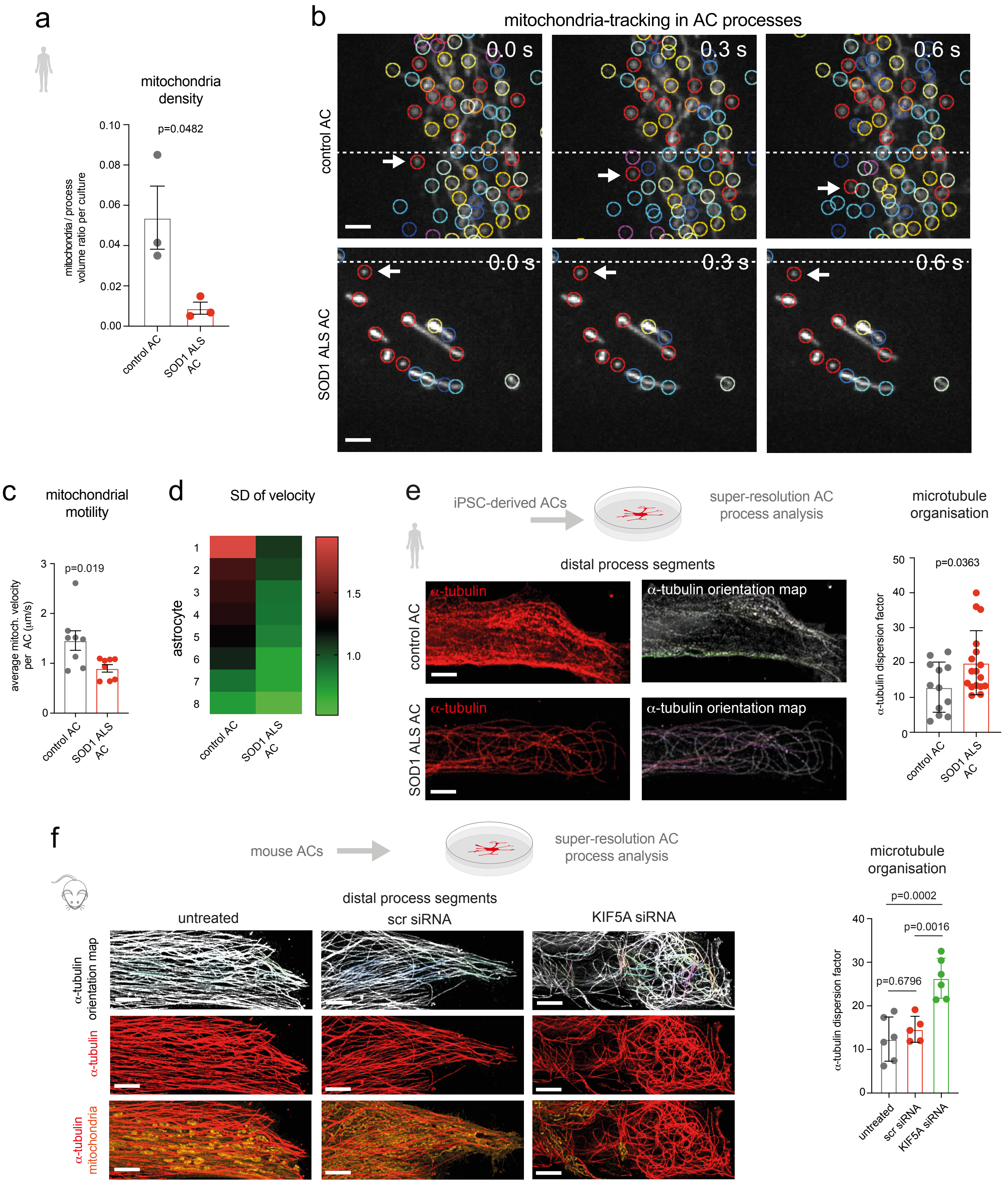

Supplementary Fig 5 Analysis of mitochondrial density, motility and microtubule organisation in control and SOD1 ALS astrocyte processes. a Graph represents mitochondrial densities (ratio of mitochondria and process volume) per AC culture. n=3 independent AC culture batches; data expressed as mean±SEM. **b** Sequential captured representative spinning disc confocal microscopy images, demonstrating the movements of MitoBright prelabelled mitochondria incontrol and ALS astrocyte (AC) processes. Coloured circles illustrate the identified mitochondria by MitoTracker (ImageJ plugin), and white arrows indicate the same mitochondrium tracked through 0.6 seconds either in control or ALS ACs. Dotted line represents the reference position of tracked mitochondria in the y-axis at the start of the recording. **c** Plots represent the average mitochondrial velocity (µm/sec) in processes in the two groups, and the bars indicate the mean values for ACs per group. n=8 AC per group; two-tailed unpaired t-test; data is expressed as mean±SEM. **d** Heatmap illustrating the standard deviation (SD) of mitochondrial velocity in each AC (bright red=higher SD and light green=low SD). **e** Representative superresolution structured illumination microscopy (SR-SIM) immunofluorescence images of control and ALS AC processes (distal 20µm segment), demonstrating a-tubulin immunoreactivity (left) and a-tubulin+ microtubule orientation maps (right) generated in the "directionality" plugin in ImageJ. Graph represents dispersion factor values as a measure of microtubule organisation. n=12 and 17 for control and ALS ACs, respectively; two-tailed unpaired t-test; data expressed as mean±SD. **f** Representative SR-SIM images of untreated, scr siRNA-treated and KIF5A siRNA-treated mouse AC cultures, illustrating the distal 20µm process segments. Graph represents dispersion factor values. n=6,5,6 untreated, scr siRNA-treated and KIF5A siRNAtreated ACs, respectively; one-way ANOVA with Tukey's posthoc test; data is expressed mean±SD. Scale bar: 2µm for b and e, 3µm for f. Supplementary information for Fig. 5.

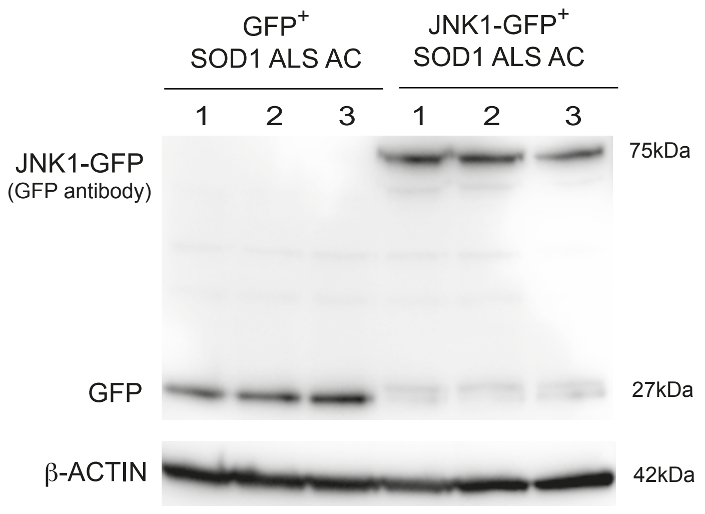

Supplementary Fig 6 Transfected SOD1 ALS astrocytes display JNK-GFP fusion protein expression. GFP western blot images demonstrating immunoreactive bands for the GFP protein and for the JNK1-GFP fusion protein in separately transfected SOD1 ALS astrocyte cultures (n=3 independently transfected cultures per group). Supplementary information for Fig. 6a.

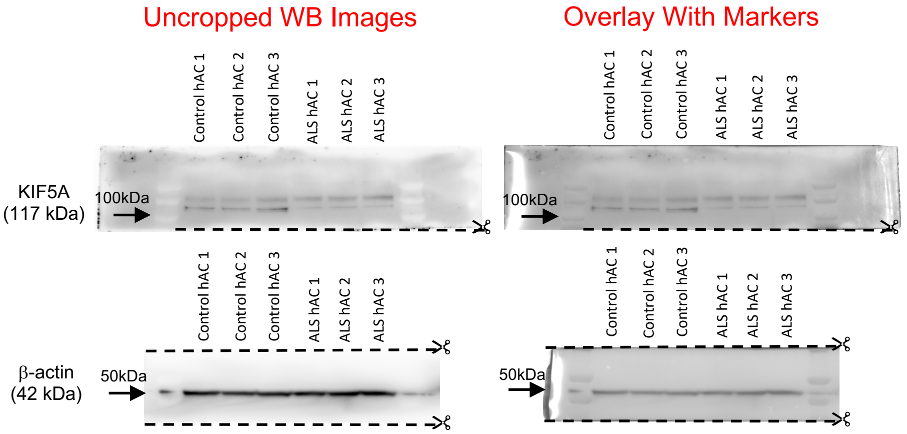

Supplementary Fig 7 Uncropped western blot (WB) images (left; luminescence channel) and their overlays with the corresponding images of molecular weight markers (right; light channel) for Fig. 3d. Sample labels refer to human astrocyte cultures (hAC) followed by batch codes. Dashed lines indicate blot membrane cuts that enabled simultaneous detection of multiple proteins in the same sample.

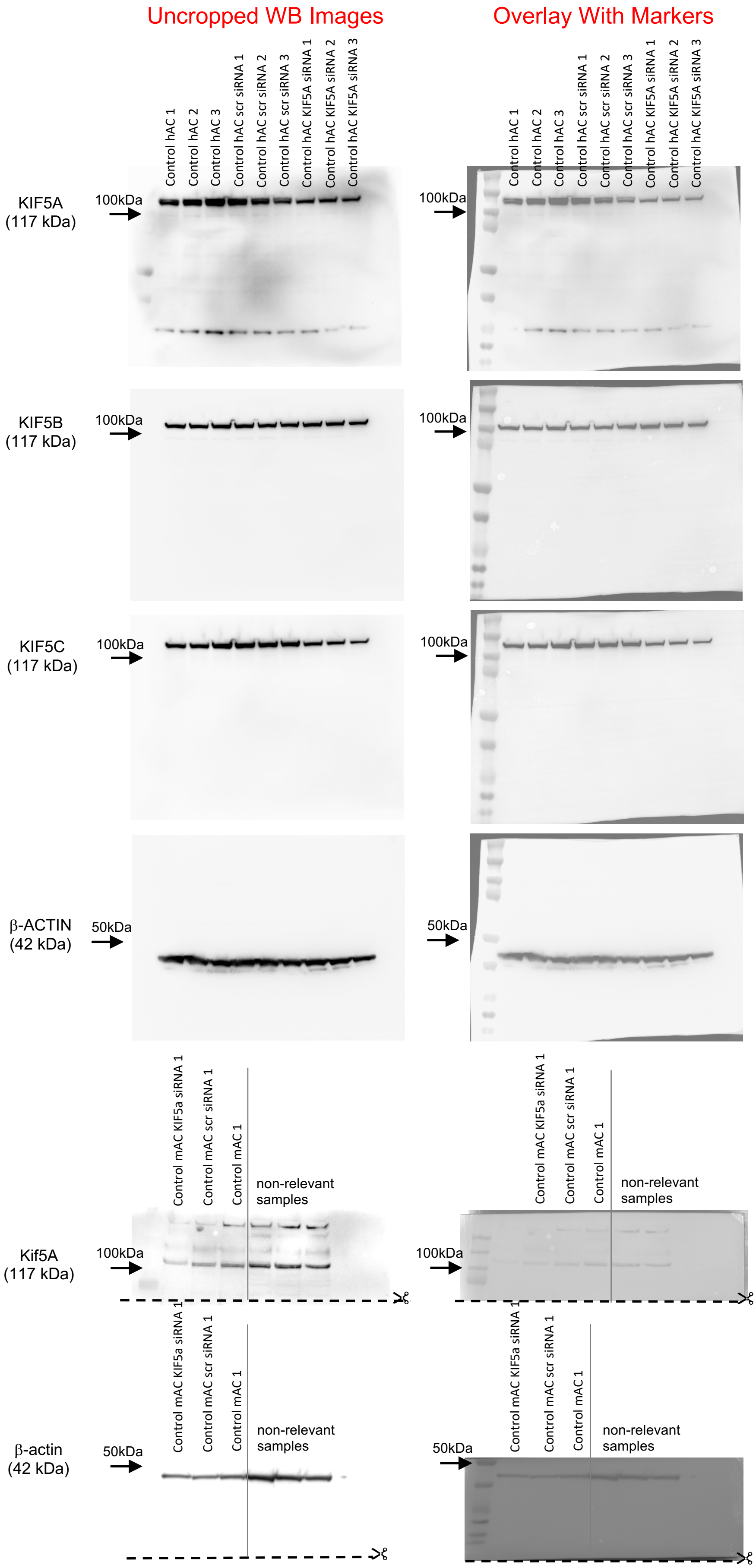

Supplementary Fig 8 Uncropped western blot (WB) images (left; luminescence channel) and their overlays with the corresponding images of molecular weight markers (right; light channel) for Fig. 4a,e. Sample labels refer to human astrocyte cultures (hAC) followed by batch codes. Dashed lines indicate blot membrane cuts that enabled simultaneous detection of multiple proteins in the same sample. Samples that are not related to this work are labelled as ”non-relevant samples”.

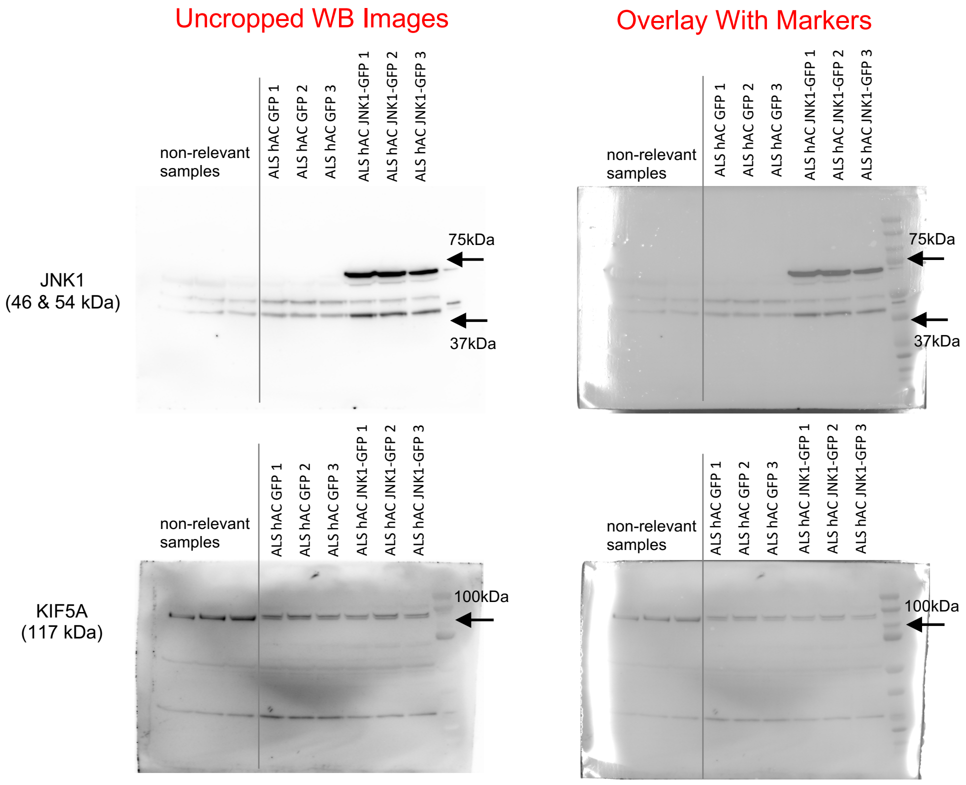

Supplementary Fig 9 Uncropped western blot (WB) images (left; luminescence channel) and their overlays with the corresponding images of molecular weight markers (right; light channel) for Fig. 6a. Sample labels refer to human astrocyte cultures (hAC) followed by batch codes. Samples that are not related to this work are labelled as ”non-relevant samples”.

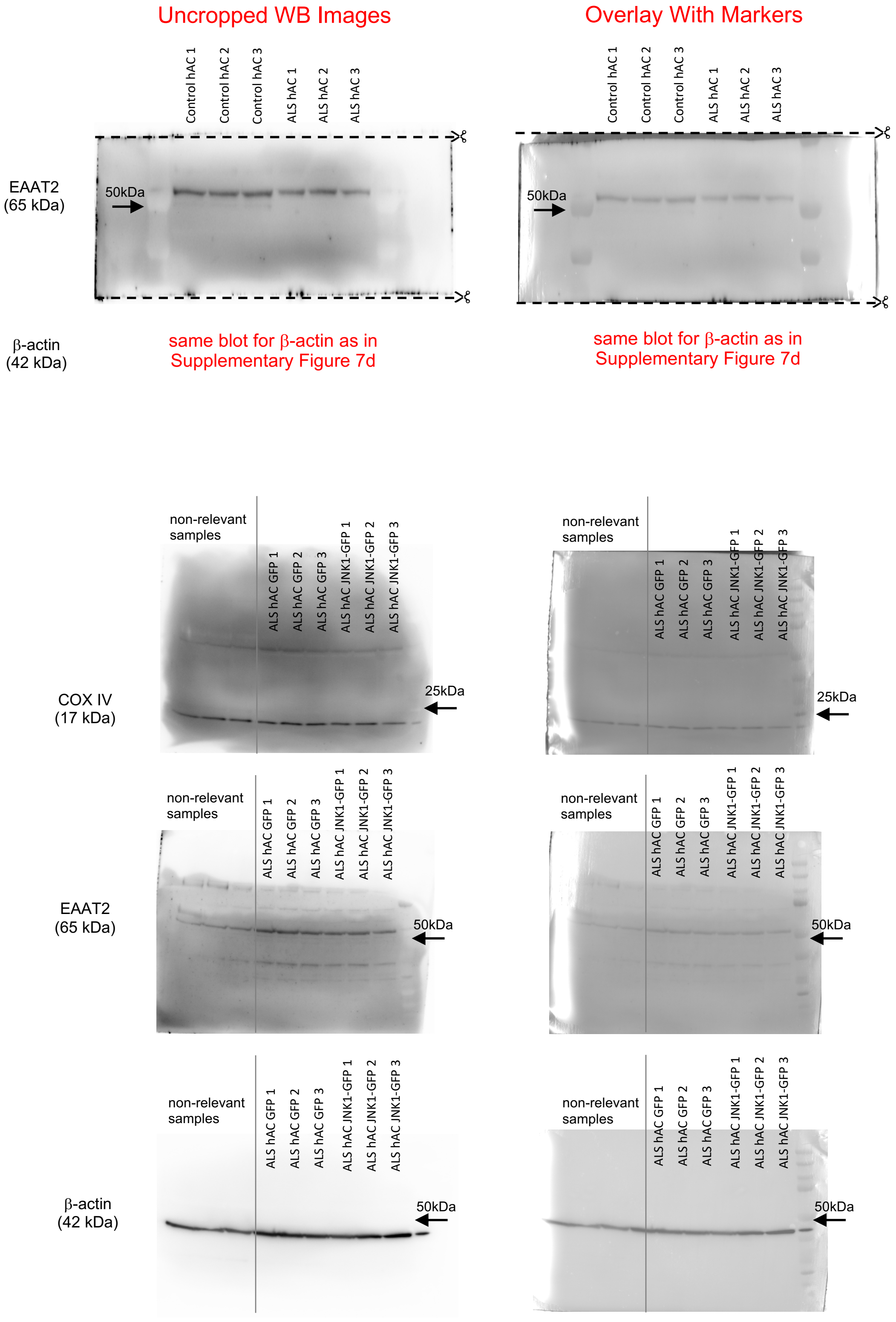

Supplementary Fig 10 Uncropped western blot (WB) images (left; luminescence channel) and their overlays with the corresponding images of molecular weight markers (right; light channel) for Fig. 7c,f. Sample labels refer to human astrocyte cultures (hAC) followed by batch codes. Dashed lines indicate blot membrane cuts that enabled simultaneous detection of multiple proteins in the same sample. Samples that are not related to this work are labelled as "non-relevant samples".

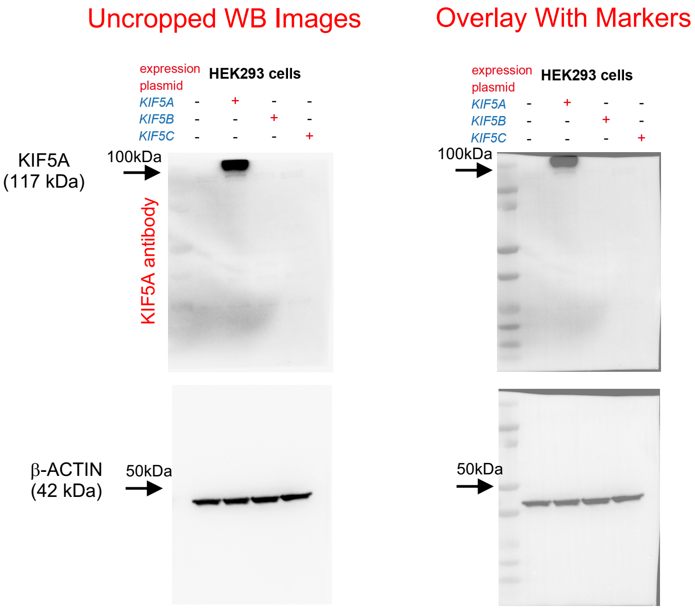

Supplementary Fig 11 Uncropped western blot (WB) images (left; luminescence channel) and their overlays with the corresponding images of molecular weight markers (right; light channel) for Suppl. Fig. 4a. Sample labels refer to human astrocyte cultures (hAC) followed by batch codes.

Supplementary Table 1 Details of human postmortem samples and iPSC lines

ID	Mutation	Age	Sex	Source ID	Sample Detail	Licenses/MTA
**ALS-1**	SOD1 (L8V)	61	F	MRC BBN_16392	lumbar spinal cord	HTA_12293<br>Bank Ethics_18/WA/0206
**ALS-2**	SOD1 (I113T)	70	F	MRC BBN_6209	lumbar spinal cord	HTA_12293<br>Bank Ethics_18/WA/0206
**ALS-3**	SOD1 (D101G)	46	F	MRC BBN_16553	lumbar spinal cord	HTA_12293<br>Bank Ethics_18/WA/0206
**ASL-4 line**	SOD1 (D90A)	70	F	NINDS ND35664	hiPSC (from fibroblasts)	MTA_G107192 (for AL)
**Control-1**	None	58	F	MRC BBN_15629	lumbar spinal cord	HTA_12293<br>Bank Ethics_18/WA/0206
**Control-2**	None	62	M	MRC BBN_16256	lumbar spinal cord	HTA_12293<br>Bank Ethics_18/WA/0206
**Control-3**	None	51	F	MRC BBN_4193	lumbar spinal cord	HTA_12293<br>Bank Ethics_18/WA/0206
**Control--4 line**	None	54	F	EBiSC WTSIi097-A	hiPSC (from fibroblasts)	MTA_RG92224 (for AL)

Supplementary Table 2 List of antibodies used

Primary Antibodies	Source and Catalogue Number	Working Dilution	Species
**α-TUBULIN**	Sigma-Aldrich T6199-100UL	1:800 for ICC	Mouse
**ACTB-HRP**	Proteintech HRP-60008	1:10000 for WB	Mouse
**KIF5A**	Abcam ab5628	1:200 for ICC	Rabbit
**KIF5A**	Abcam ab5628	1:1000 for WB	Rabbit
**KIF5B**	Abcam ab167429	1:1000 for WB	Rabbit
**KIF5C**	Abcam ab193352	1:1000 for WB	Rabbit
**JNK1**	Proteintech 24164-1-AP	1:1000 for WB	Rabbit
**COX IV**	Cell Signaling Technology 4850	1:1000 for WB	Rabbit
**EAAT2**	Cell Signaling Technology 20848S	1:200 for ICC	Rabbit
**EAAT2**	Cell Signaling Technology 20848S	1:1000 for WB	Rabbit
**ALDH1L1**	Abcam ab190298	1:200 for ICC	Rabbit
**GFAP**	Abcam ab7260	1:500 for ICC	Rabbit
**GFAP**	Antibodies.com A85307	1:800 for ICC	Chicken
**GFP**	Proteintech 50430-2-AP	1:1000 for WB	Rabbit
**tRFP (for mScarlet)**	Evrogen AB233	1:500 for ICC	Rabbit

Secondary Antibodies	Source and Catalogue Number	Working Dilution	Species
**Anti-Rabbit IgG-HRP**	ThermoFisher 31462	1:10000 for WB	Goat
**Anti-Mouse IgG-HRP**	Vector Laboratories Inc. PI-2000	1:10000 for WB	Goat
**Anti-Chicken Alexa 405**	Abcam ab175674	1:500 for ICC	Goat
**Anti-Rabbit Alexa Fluor-568**	ThermoFisher A11036	1:500 for ICC	Goat
**Anti-Chicken Alexa Fluor-647**	ThermoFisher A32933	1:500 for ICC	Goat
**Anti-Mouse Alexa Fluor-647**	Abcam ab150119	1:500 for ICC	Goat

Supplementary Table 3 Details of statistical tests and sample sizes

figure number	statistical test	n-values and sample size details	data representation	groups	p-value	further statistical details
**Fig. 1b**	Receiver operating characteristic (ROC) curves	Sample size (foreground/background)<br>ALS: 10/17970 (max), 31/17949 (sco>Q3), 76/17904 (ChEMBL)<br>PD: 15/17924 (max), 54/17885 (sco>Q3), 123/17816 (ChEMBL)<br>AD: 12/17563 (max), 61/17514 (sco>Q3), 117/17458 (ChEMBL)	area under de ROC curve (AUC)			ALS AUCs: 0.8685 (max),0.8311 (sco>Q3),0.7347 (ChEMBL)<br>PD AUCs: 0.7361 (max),0.7137 (sco>Q3),0.7083 (ChEMBL)<br>AD AUCs: 0.7333 (max), 0.6817 (sco>Q3), 0.7006(ChEMBL)
**Fig. 2c**	Fisher exact test (one sided) plus multiple testing corrections using the Benjamini-Hochberg adjustment	Ratios for the foreground (GO term vs total genes in the module): 91/233, 89/233, 93/233, 93/233, 95/233<br>Ratios for the background (sGO term vs total genes in the module): 99/16174, 95/16174, 108/16174, 112/16174, 120/16174.	-log10(q-value)	7.35E-166, 4.77E-164, 2.74E-163, 4.25E-160,<br>adjusted p-values: 1.28E-162 4.14E-161 1.59E-160		
**Fig. 3c**	Two-tailed unpaired t-test	3 ALS patient-derived postmortem spinal cord tissue samples (harbouring SOD1 mutations; 3 sections per sample)	mean ± s.e.m.	control vs ALS spinal cord	0.0008	t=9.25, df=4
**Fig. 3d**	Two-tailed unpaired t-test	3 independently differentiated cultures (batches) of healthy and ALS astrocytes	mean ± s.e.m.	control vs ALS astrocytes	0.0085	t=4.817, df=4
**Fig. 3e**	Two-tailed unpaired t-test	4 independently differentiated healthy and 5 ALS astrocyte cultures (healthy: 39, 34, 50, 43; ALS: 59, 10, 42, 49, 29 astrocytes analyzed per culture)	mean ± s.e.m.	control vs ALS astrocytes	0.0006	t=5.855, df=7
**Fig. 4b**	One way ANOVA, Tukey's posthoc test	3 independently transfected cultures of healthy astrocytes	mean ± s.e.m.	untreated vs scr siRNA<br>untreated vs KIF5A siRNA<br>scr siRNA vs KIF5A shRNA	0.6594<br>0.0151<br>0.0428	F(2,6)=9.263, p=0.0146
		3 independently transfected cultures of healthy astrocytes	mean ± s.e.m.			F(2,6)=4.790, p=0.0571
		3 independently transfected cultures of healthy astrocytes	mean ± s.e.m.			F(2,6)=1.402, p=0.3165
**Fig. 4c**	Two-tailed unpaired t-test	4 independently transfected cultures of healthy astrocytes by scr shRNA and 5 independently transfected cultures of healthy astrocytes by KIF5A shRNA (scr shRNA: 25, 21, 32, 33; KIF5A shRNA: 27, 22, 23, 23, 21 astrocytes analyzed per culture)	mean ± s.e.m.	scr siRNA vs KIF5A siRNA	0.032	t=2.669, df=7
**Fig. 4d**	One way ANOVA, Tukey's posthoc test	3 independently transfected cultures of control and ALS astrocytes (79 non-transfected, 54 transfected control astrocytes and 37 non-transfected, 19 transfected ALS astrocytes)	mean ± s.e.m.	mScarlet- vs mScarlet+ control astrocytes<br>mScarlet- control vs mScarlet- ALS astrocytes<br>mScarlet- control vs mScarlet+ ALS astrocytes<br>mScarlet+ control vs mScarlet- ALS astrocytes<br>mScarlet+ control vs mScarlet+ ALS astrocytes<br>mScarlet- vs mScarlet+ ALS astrocytes	0.2567<br>0.0144<br>0.1818<br>0.0013<br>0.9934<br>0.001	F (3, 8) = 17.45, p=0.0007
**Fig. 4e**	Two-tailed unpaired t-test	3 independently transfected cultures of wild type mouse (C57Bl/6) astrocytes (6 mice per culture)	mean ± s.e.m.	scr siRNA vs KIF5A siRNA	0.0064	t=5.233, df=4
**Fig. 4f**	Two-tailed unpaired t-test	5 independently transfected cultures of healthy astrocytes by scr shRNA and 5 ndependently transfected cultures of healthy astrocytes by KIF5A shRNA (6 mice/culture; scr shRNA: 10, 12, 13, 8, 6; Kif5a shRNA: 19, 18, 18, 5, 6 astrocytes analyzed per culture)	mean ± s.e.m.	scr siRNA vs KIF5A siRNA	0.0469	t=2.347, df=8
**Fig. 5b**	Two-tailed unpaired t-test	22 control and 21 SOD1 ALS astrocyte processes for super-resolution microscopy analyses; KIF5A+ particle density readings at 1μm intervals (distal 15μm process length) from 3 cultures.	mean ± s.e.m.	control vs ALS astrocytes	<0.0001	t=7.01, df=28
**Fig. 5c**	Two-tailed Mann Whitney test	21 control and 22 SOD1 ALS astrocyte processes for super-resolution microscopy analyses (from 3 cultures).	mean ± s.d.	control vs ALS astrocytes	<0.0001	MW U=35
**Fig. 5e**	Two-tailed unpaired t-test	7 control scr siRNA-treated and 7 KIF5A siRNA-treated control astrocyte processes for super-resolution microscopy analyses; KIF5A+ particle density readings at 1μm intervals.	mean ± s.e.m.	scr siRNA vs KIF5A siRNA	0.0005	t=3.96, df=28
**Fig. 5f**	Two-tailed Mann Whitney test	8 control scr siRNA-treated and 8 KIF5A siRNA-treated control astrocyte processes for super-resolution microscopy analyses	mean ± s.d.	scr siRNA vs KIF5A siRNA	0.0019	MW U=4
**Fig. 6a**	Two-tailed unpaired t-test	For JNK1 WB: 3 independently transfected cultures of ALS astrocytes	mean ± s.e.m.	GFP vs JNK1-GFP astrocytes	0.0065	t=5.205, df=4
		For KIF5A WB: 3 independently transfected cultures of ALS astrocytes			0.5795	t=0.6023, df=4
**Fig. 6b**	Two-tailed unpaired t-test	3 independently transfected cultures of SOD1 ALS astrocytes expressing GFP and 5 independently transfected cultures of SOD1 ALS astrocytes expressing JNK-GFP	mean ± s.e.m.	JNK1-GFP- vs JNK1-GFP+	<0.0001	t=10.32, df=6
**Fig. 6d**	One way ANOVA, Tukey's posthoc test	6 GFP+, 18 JNK-GFP+ and 8 JNK-GFP- astrocyte processes for super-resolution microscopy based analyses; KIF5A+ particle density readings at 1μm intervals.	mean ± s.e.m.	overall ANOVA	0.0002	F (2, 42) = 10.92
			line = median<br>box = upper and lower quartiles<br>whiskers = 5-95 percentile	GFP vs JNK-GFP<br>GFP vs non-JNK1-GFP<br>JNK-GFP vs non-JNK1-GFP	0.0034<br>0.5941<br>0.0002	
**Fig. 7b**	Two-tailed unpaired t-test	8 control and 8 SOD1 ALS astrocytes analyzed (5-6 images per astrocyte) from two experiments	mean ± s.d.	control vs ALS astrocytes	<0.0001	t=4.90, df=14
**Fig. 7c**	Two-tailed unpaired t-test	3 indepenent batches of control and SOD1 ALS astrocyte cultures	mean ± s.e.m.	control vs ALS astrocytes	0.0087	t=4.788, df=4
**Fig. 7e**	Two-tailed unpaired t-test	8 JNK-GFP- and 10 JNK-GFP+ SOD1 ALS astrocytes analyzed (5-6 images per astrocyte) from two experiments	mean ± s.d.	JNK1-GFP- vs JNK1-GFP+	<0.0001	t=4.51, df=16
**Fig. 7f**	Two-tailed unpaired t-test	For COX IV: 3 independently transfected cultures of ALS astrocytes by either GFP or JNK-GFP vectors	mean ± s.e.m.	GFP vs JNK1-GFP ALS astrocytes	0.8122	t=0.2538, df=4
		For EAAT2: 3 independently transfected cultures of ALS astrocytes by either GFP or JNK-GFP vectors			0.398	t=0.9455, df=4
