# Supplementary material for: A human proteogenomic-cellular framework identifies KIF5A as a modulator of astrocyte process integrity with relevance to ALS
Source: Commun Biol. 2023 Jun 29;6:678. doi: 10.1038/s42003-023-05041-4 (PMC10310856; doi:10.1038/s42003-023-05041-4)
Supplement: Supplementary file 2 — Description of Additional Supplementary Files [file 42003_2023_5041_MOESM2_ESM.pdf]

Description of Additional Supplementary Files

File name: Supplementary Data 1
Description: The source data behind Figure 1

File name: Supplementary Data 2
Description: The source data behind Figure 2

File name: Supplementary Data 3
Description: The source data behind Figure 3

File name: Supplementary Data 4
Description: The source data behind Figure 4

File name: Supplementary Data 5
Description: The source data behind Figure 5

File name: Supplementary Data 6
Description: The source data behind Figure 6

File name: Supplementary Data 7
Description: The source data behind Figure 7
