# Supplementary material for: A human proteogenomic-cellular framework identifies KIF5A as a modulator of astrocyte process integrity with relevance to ALS
Source: Commun Biol. 2023 Jun 29;6:678. doi: 10.1038/s42003-023-05041-4 (PMC10310856; doi:10.1038/s42003-023-05041-4)
Supplement: Supplementary file 10 — Reporting Summary [file 42003_2023_5041_MOESM10_ESM.pdf]

nature portfolio

Corresponding author(s): Dr András Lakatos

Last updated by author(s): Jun 4, 2023

Reporting Summary

Nature Portfolio wishes to improve the reproducibility of the work that we publish. This form provides structure for consistency and transparency in reporting. For further information on Nature Portfolio policies, see our Editorial Policies and the Editorial Policy Checklist.

Statistics

For all statistical analyses, confirm that the following items are present in the figure legend, table legend, main text, or Methods section.

n/a	Confirmed	
☐	☒	The exact sample size (*n*) for each experimental group/condition, given as a discrete number and unit of measurement
☐	☒	A statement on whether measurements were taken from distinct samples or whether the same sample was measured repeatedly
☐	☒	The statistical test(s) used AND whether they are one- or two-sided<br>*Only common tests should be described solely by name; describe more complex techniques in the Methods section.*
☒	☐	A description of all covariates tested
☐	☒	A description of any assumptions or corrections, such as tests of normality and adjustment for multiple comparisons
☐	☒	A full description of the statistical parameters including central tendency (e.g. means) or other basic estimates (e.g. regression coefficient) AND variation (e.g. standard deviation) or associated estimates of uncertainty (e.g. confidence intervals)
☐	☒	For null hypothesis testing, the test statistic (e.g. *F*, *t*, *r*) with confidence intervals, effect sizes, degrees of freedom and *P* value noted<br>*Give P values as exact values whenever suitable.*
☒	☐	For Bayesian analysis, information on the choice of priors and Markov chain Monte Carlo settings
☒	☐	For hierarchical and complex designs, identification of the appropriate level for tests and full reporting of outcomes
☒	☐	Estimates of effect sizes (e.g. Cohen's *d*, Pearson's *r*), indicating how they were calculated

Our web collection on statistics for biologists contains articles on many of the points above.

Software and code

Policy information about availability of computer code

Data collection	For image capturing the following softwares were used:<br><br>Zeiss ZEN software (built in; Zeiss Elyra PS1)<br>https://www.zeiss.com/microscopy/int/products/microscope-software/zen.html<br>Leica Application Suite (LAS, built in; Leica DM6000)<br>https://www.leica-microsystems.com/products/light-microscopes/p/leica-dm6000-m/gallery/
Data analysis	Established open source softwares, plugins or pipelines were used for data analysis, and the details have been provided in the manuscript and also below with full references and/or web links for accessibility. No new codes were developed for data analysis in this study.<br><br>CellProfiler Image Analysis Software, 3.1. 9 and 4.0.7, RRID: SCR_007358, https://cellprofiler.org/<br>FIJI (ImageJ), 2.0.0, RRID: SCR_002285, https://fiji.sc/<br>GraphPad, Version 7.0 or 8.0, RRID:SCR_002798, https://www.graphpad.com/<br>R version 4.0.3, RRID:SCR_001905, https://www.r-project.org

For manuscripts utilizing custom algorithms or software that are central to the research but not yet described in published literature, software must be made available to editors and reviewers. We strongly encourage code deposition in a community repository (e.g. GitHub). See the Nature Portfolio guidelines for submitting code & software for further information.

Data

Policy information about availability of data

All manuscripts must include a data availability statement. This statement should provide the following information, where applicable:

- Accession codes, unique identifiers, or web links for publicly available datasets
- A description of any restrictions on data availability
- For clinical datasets or third party data, please ensure that the statement adheres to our policy

Data that support the results in this work are provided as Source Files (Source Data File 1 for Figure 1, Source Data File 2 for Figure 2) or can be found in the Supplementary Figures. Data are also available from the corresponding authors upon request.

Human research participants

Policy information about studies involving human research participants and Sex and Gender in Research.

Reporting on sex and gender	Our integrative GWAS catalog-multiomics network analysis was performed on published datasets based on anonymous and sex-matched datasets. Mechanistic experiments and validations were performed using female control and ALS patient lines and postmortem samples deriving mostly from female patients. No personal patient data were available for the investigators.
Population characteristics	Samples were age-matched
Recruitment	Human brain tissue samples were obtained from the London Neurodegenerative Diseases Brain Bank at King's College. Human cell lines were obtained from commercial sources (European Bank for Induced pluripotent Stem Cells (EBiSC) and the National Institute of Neurological Disorders and Stroke (NINDS) repository.
Ethics oversight	London Neurodegenerative Diseases Brain Bank at King's College, London by C.T. (HTA licence reference: 12293; human tissue bank ethics reference: 18/WA/0206; consent provided by the Brain Bank).

Note that full information on the approval of the study protocol must also be provided in the manuscript.

Field-specific reporting

Please select the one below that is the best fit for your research. If you are not sure, read the appropriate sections before making your selection.

☒ Life sciences ☐ Behavioural & social sciences ☐ Ecological, evolutionary & environmental sciences

For a reference copy of the document with all sections, see nature.com/documents/nr-reporting-summary-flat.pdf

Life sciences study design

All studies must disclose on these points even when the disclosure is negative.

Sample size	Statistical test details and sample sizes have been summarised in Supplementary Table 3. No sample size calculation was performed. The sample sizes using hiPSC lines were estimated from previously performed experiments (e.g. Tyzack et al., Nature communications, 2017).
Data exclusions	No acquired data was excluded from the statistical analyses. (Note: 2 damaged histological sections and 2 images of astrocyte processes could not used for data collection due to imaging artefacts).
Replication	Experiments were repeated three times (or two times for Super-Resolution Microscopy due to low-throughput limitations of this approach), which included at least 3 independent biological replicates (independent culture batches/transfections). At least three biological replicates were used for statistical analyses in biological experiments, except when plotting and comparing the variance of object distributions across individual astrocyte processes/cells were more informative than the comparison of averaged values per culture (Figure 5b,c,e,f; Figure 6d, Figure 7b-right graph, 7e).
Randomization	Human postmortem spinal cord tissue samples were randomly selected from the Brain Bank for both ALS and control groups. For in vitro experiments, no randomization was performed.
Blinding	For analysis, the subjects were blinded for the observers. This involved masking original sample identification and assigning coded IDs before data collection.

Reporting for specific materials, systems and methods

We require information from authors about some types of materials, experimental systems and methods used in many studies. Here, indicate whether each material, system or method listed is relevant to your study. If you are not sure if a list item applies to your research, read the appropriate section before selecting a response.

Materials & experimental systems

n/a	Involved in the study
☐	☒ Antibodies
☐	☒ Eukaryotic cell lines
☒	☐ Palaeontology and archaeology
☐	☒ Animals and other organisms
☒	☐ Clinical data
☒	☐ Dual use research of concern

Methods

n/a	Involved in the study
☒	☐ ChIP-seq
☒	☐ Flow cytometry
☒	☐ MRI-based neuroimaging

Antibodies

Antibodies used

The details of all antibodies are provided below with validation references, and are also listed in Supplementary Table 2.

Validation

Antibody details, catalogue number, validation reference:

Mouse anti-alpha-tubulin, Sigma-Aldrich T6199
RRID: AB_477583
Mouse anti-ACTB-HRP, Proteintech HRP-60008
RRID:AB_2819183
Rabbit anti-KIF5A, Abcam ab5628
RRID: AB_2132218
Rabbit anti-KIF5B, Abcam ab167429
RRID: AB_2715530
Rabbit anti-KIF5C, Abcam ab193352
https://www.abcam.com/products/primary-antibodies/kif5c-antibody-epr16224-32-ab193352.html
Rabbit anti-JNK1, Proteintech 24164-1-AP
RRID: AB_2879443
Rabbit anti-COX IV, Cell Signaling Technology 4850
RRID: AB_2085424
Rabbit anti-EAAT2, Cell Signaling Technology 20848S
https://www.cellsignal.com/products/primary-antibodies/eaat2-e3p5k-rabbit-mab/20848
Rabbit anti-ALDH1L1, Abcam ab190298
RRID: AB_2857848
Rabbit anti-GFAP, Abcam ab7260
https://www.abcam.com/gfap-antibody-ab7260.html
Chicken anti-GFAP, Antibodies.com A85307
RRID:AB_2748894
Rabbit anti-GFP, Proteintech 50430-2-AP
https://www.ptglab.com/products/eGFP-Antibody-50430-2-AP.htm
Rabbit anti-RFP, Evrogen AB233
RRID:AB_2571743

Goat anti-Rabbit IgG-HRP, ThermoFisher 31462
RRID:AB_228338
Goat anti-Mouse IgG-HRP, Vector Laboratories Inc. PI-2000
https://vectorlabs.com/peroxidase-horse-anti-mouse-igg-antibody.html#biozbadges
Goat anti-Chicken Alexa 405, Abcam ab175674
RRID: AB_2890171
Goat anti-Rabbit Alexa Fluor-568, ThermoFisher A11036
RRID:AB_10563566
Goat anti-Chicken Alexa Fluor-647, ThermoFisher A32933
RRID:AB_2762845
Goat anti-Mouse Alexa Fluor-647, Abcam ab150119
RRID: AB_2811129

Eukaryotic cell lines

Policy information about cell lines and Sex and Gender in Research

Cell line source(s)

All cell line sources and details are listed in Supplementary Table 1.

Authentication

The genotyping was performed by repositories where the cell lines (NINDS) and human tissue (London Neurodegenerative Diseases Brain Bank, King's College) were obtained from.

Mycoplasma contamination	Media from cell lines were randomly tested for Mycoplasma in our laboratory, and there has been no evidence for contamination by Mycoplasma.
Commonly misidentified lines (See ICLAC register)	No commonly misidentified cell lines were used.

Animals and other research organisms

Policy information about studies involving animals; ARRIVE guidelines recommended for reporting animal research, and Sex and Gender in Research

Laboratory animals	Mixed gender newborn pups were used for the generation of mouse astrocyte cultures. The schedule 1 protocol was used by authorized personnel for culling and for the retrieval of spinal cord and brainstem tissue.
Wild animals	None
Reporting on sex	Mixed female/male mouse pups were used to generate astrocyte cultures, which did not allow reporting on sex-dependent observations.
Field-collected samples	None
Ethics oversight	Mouse pups (P0-P1) were culled by authorised laboratory personnel and mouse tissue was according the regulations by the UK Home Office and local laboratory guidance.

Note that full information on the approval of the study protocol must also be provided in the manuscript.
